# A chromosome-level genome of the booklouse, *Liposcelis brunnea*, provides insight into louse evolution and environmental stress adaptation

**DOI:** 10.1093/gigascience/giac062

**Published:** 2022-07-19

**Authors:** Shiqian Feng, George Opit, Wenxin Deng, Vaclav Stejskal, Zhihong Li

**Affiliations:** Department of Plant Biosecurity, College of Plant Protection, China Agricultural University, Beijing 100193, China; Key Laboratory of Surveillance and Management for Plant Quarantine Pests, Ministry of Agriculture and Rural Affairs, Beijing 100193, China; Department of Entomology and Plant Pathology, Oklahoma State University, Oklahoma 74078, Stillwater, USA; Department of Plant Biosecurity, College of Plant Protection, China Agricultural University, Beijing 100193, China; Key Laboratory of Surveillance and Management for Plant Quarantine Pests, Ministry of Agriculture and Rural Affairs, Beijing 100193, China; Crop Research Institute, Drnovská 507, 161 06 Prague 6, Czech Republic; Faculty of Agrobiology, Food and Natural Resources, Czech University of Life Sciences, Kamycka 129, 165 00 Prague, Czech Republic; Department of Plant Biosecurity, College of Plant Protection, China Agricultural University, Beijing 100193, China; Key Laboratory of Surveillance and Management for Plant Quarantine Pests, Ministry of Agriculture and Rural Affairs, Beijing 100193, China

**Keywords:** booklice, Liposcelis brunnea, genome assembly, louse evolution, insecticide resistance, high temperature tolerance

## Abstract

**Background:**

Booklice (psocids) in the genus *Liposcelis* (Psocoptera: Liposcelididae) are a group of important storage pests, found in libraries, grain storages, and food-processing facilities. Booklice are able to survive under heat treatment and typically possess high resistance to common fumigant insecticides, hence posing a threat to storage security worldwide.

**Results:**

We assembled the genome of the booklouse, *L. brunnea*, the first genome reported in Psocoptera, using PacBio long-read sequencing, Illumina sequencing, and chromatin conformation capture (Hi-C) methods. After assembly, polishing, haplotype purging, and Hi-C scaffolding, we obtained 9 linkage groups (174.1 Mb in total) ranging from 12.1 Mb to 27.6 Mb (N50: 19.7 Mb), with the BUSCO completeness at 98.9%. In total, 15,543 genes were predicted by the Maker pipeline. Gene family analyses indicated the sensing-related gene families (OBP and OR) and the resistance-related gene families (ABC, EST, GST, UGT, and P450) expanded significantly in *L. brunnea* compared with those of their closest relatives (2 parasitic lice). Based on transcriptomic analysis, we found that the CYP4 subfamily from the P450 gene family functioned during phosphine fumigation; HSP genes, particularly those from the HSP70 subfamily, were upregulated significantly under high temperatures.

**Conclusions:**

We present a chromosome-level genome assembly of *L. brunnea*, the first genome reported for the order Psocoptera. Our analyses provide new insights into the gene family evolution of the louse clade and the transcriptomic responses of booklice to environmental stresses.

## Introduction

Psocids are stored-product arthropods that are of increasing economic importance as pests of seeds, raw agricultural materials, food, and feed [1–4]. Booklice in the genus *Liposcelis* are the most important clade across the psocids because of their global distribution and high resistance to insecticides and fumigants [5]. More generally, booklice were known as minute, pale insects found scuttling across books or stacks of papers [6]. Booklice infestations are usually a result of poor storage conditions associated with high moisture, which negatively influences the commodity [7]. Psocids feeding can cause a 5–10% weight loss in agricultural commodities [8, 9]. They can also have negative impacts on human health through the production of allergens [10, 11] or transmission of parasites [12].

Contact insecticides and fumigants are used for managing booklice. However, booklice can develop insecticide resistance compared with other stored product pests [2]. For example, deltamethrin, carbaryl, and methoprene, which can control beetles and moths, are not effective against booklice [13, 14]. Moreover, booklice are documented to possess high resistance to bacterium-derived spinosad, imidacloprid, and diatomaceous earth [15, 16]. Phosphine fumigation is the most popular method for managing storage pests [17]. A high level of resistance to phosphine has been observed in booklice, particularly during the egg stage, which significantly increases both the economic cost of treatments and environmental pollution [18, 19]. Several gene families have been proven to be related to the high insecticide resistance of booklice, including esterases (ESTs) [20], glutathione S-transferase (GST) [21], and the cytochrome P450 monooxygenases (P450) [22]. These studies were mainly based on transcriptomic analyses and lacked whole-genome data. We know that in certain circumstances, mRNA analysis can be influenced by insect age or other factors. These factors can also affect gene family evaluation by eliminating those genes with low expression [23]. As a result, it is necessary to develop a nuclear genome of booklice species for resistance-related analyses.

Booklice are the phylogenetic sister group to parasitic lice and they have been considered a key taxon in determining the origins and evolution of parasitic lice [24–26]. The habits of *Liposcelis* species are similar to those of parasitic lice; for example, they are found in the nests of birds and mammals, indicating a close relationship with their potential host [27–29]. Therefore, identifying the shared features of booklice and parasitic lice, particularly their genome features, could provide unique insight into the origin of parasitism. To date, only 2 parasitic genomes have been published, and they present conflicting results with regard to the detoxification- and sensing-related gene families [30, 31]. Uncovering a booklice genome thus could help resolve related questions.

**Figure 1.**
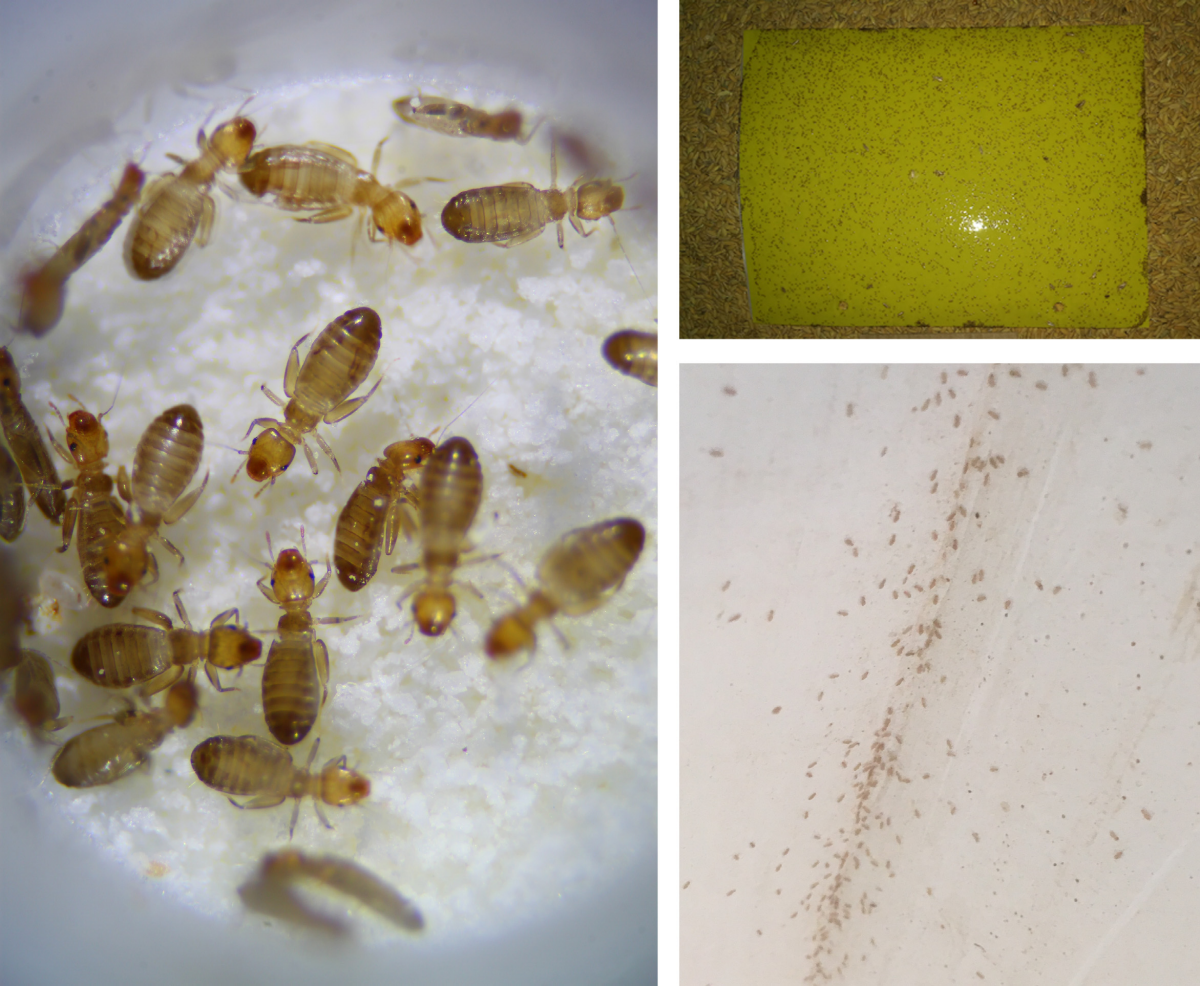
The adult booklouse, *Liposcelis brunnea* in the present study. They are reared on an artificial diet in the lab (left). They are always trapped in the grain storage, especially the corner of storage facilities (right).

Here, we present a high-quality genome assembly of *Liposcelis brunnea* (NCBI:txid209926) (Fig. 1), the first chromosome-level genome assembly reported in Psocoptera. PacBio sequencing, Illumina, and HiC technology were leveraged in our study. Comparative genomics analysis provided new clues on the evolution of lice, and transcriptomic analysis revealed how booklice adapt to high temperature and insecticide treatment.

## Methods

### Sample collection

Samples of *L. brunnea* were collected in 2009 from Oklahoma State, in the United States, and maintained for more than 100 breeding generations. We found a long and curly *Se* in these samples, which is the typical morphological trait of *L. brunnea* compared with other booklice. The booklice were put in jars and fed a mixture of wheat flour, yeast, and whole milk powder in a ratio of 10:1:1. The rearing jars were put into incubators in a dark environment with a temperature of 25°C and a relative humidity of 75%.

### DNA extraction, RNA extraction, library construction, and sequencing

We knew that the close relatives of booklice possessed a sex determination of XO [32], which indicated that we could assemble the complete set of chromosomes using either males or females. Genomic DNA of 500 adult females was extracted using a Promega, Wisconsin, USA Genomic DNA Purification Kit (A1125). After the quality of the isolated DNA was assessed, a ∼20-kb library was constructed using a SMRTbell Express Template Prep Kit 2.0 (Pacific Biosciences, California, USA). The library construction included DNA shearing, damage repair, end repair, hairpin adapter ligation, and purification. After a quality control test, the SMRTbell library was obtained. The library was sequenced using a single 8-M SMRT Cell on the PacBio Sequel II platform (PacBio Sequel II System, RRID:SCR_017990). For genome survey and assembly polishing, we extracted genomic DNA from 40 adult females and constructed an Illumina sequencing library according to the manufacturer's instructions (Illumina, California, USA), which was then sequenced on the Illumina NovaSeq 6000 platform (Illumina NovaSeq 6000 Sequencing System, RRID:SCR_016387) in paired-end 150-bp mode (insert size 350 bp) for approximately 20 GB data. For genome annotation, we extracted total RNA from 40 adult females using the Tiangen, Beijing, China RNA extraction kit. After reverse transcribing mRNA into cDNA, another Illumina library was constructed and sequenced with the same parameters for approximately 6 GB data. The sequencing processes were conducted by the Berry Genomics Company, Beijing, China.

### Hi-C sequencing

Approximately 500 adult females were collected for the Hi-C experiments and subsequent sequencing. The library was constructed using the following steps: crosslinking the crashed samples with formaldehyde, digesting the DNA with MboI enzyme, filling ends and marks with biotin, ligating the resulting blunt-end fragments, purification, and random shearing of DNA into 300- to 500-bp fragments. After library construction following the manufacturer's instructions (Illumina), sequencing was performed on the Illumina NovaSeq 6000 platform in 150-bp paired-end mode for about 60 GB data. The experiments and sequencing were performed by Annoroad Gene Technology, Beijing, China.

### Genome survey, assembly, and quality assessment

Using the Illumina sequencing reads, we counted the 19-mers with Jellyfish v2.2.10 (Jellyfish, RRID:SCR_005491) [33] and evaluated the genome features using GenomeScope v2.0 (GenomeScope, RRID:SCR_017014) [34]. The PacBio CLR data were processed using Canu v2.1.1 (Canu, RRID:SCR_015880) [35] following correction (-correct), trimming (-trim), and assembly (-assemble) with the following parameters: minReadLength = 2,000, minOverlapLength = 500, corOutCoverage = 120, corMinCoverage = 2, and correctedErrorRate = 0.035. PacBio sequencing data and Illumina sequencing data were both leveraged to polish the draft genome. The PacBio sequencing data were mapped to the draft genome using pbmm2 v1.4.0 [36], after which gcpp v1.9.0 [37] with the arrow algorithm was used for assembly polishing. We then mapped the Illumina sequencing data to the gcpp-polished assembly using BWA v0.7.17 (BWA, RRID:SCR_010910) [38], and Pilon v1.23 (Pilon, RRID:SCR_014731) [39] was used to polish the second round. Because we set “correctedErrorRate” to a very low level in the Canu assembly step, the heterogeneous contigs were separated, producing redundant contigs. After assembly polishing, purge_dups v1.2.5 (purge dups, RRID:SCR_021173) [40] was used for the redundancy purge. The filtered HiC reads were aligned to the polished genome by BWA v0.7.17, which was integrated into Juicer v1.6 (Juicer, RRID:SCR_017226) [41]. Only uniquely mapped and valid paired-end reads were used for assembly by 3D-DNA v180114 [42]. Juicebox v1.11.08 (Juicebox, RRID:SCR_021172) [43] was used to manually order the scaffolds to obtain the final chromosome assembly. BUSCO v5.1.3 (BUSCO, RRID:SCR_015008) [44] was used to assess the completeness of the genome assembly based on the arthropoda_odb10 database.

### Genome annotation

RepeatModeler v2.0.1 (RepeatModeler, RRID:SCR_015027) [45] was used to build a custom *de novo* repeat library, based on which RepeatMasker v4.1.0 (RepeatMasker, RRID:SCR_012954) [46] was used to detect the repetitive elements. Genome structural annotation was conducted using the Maker pipeline v3.01.03 [47] with *ab initio* prediction, homology-based prediction, and RNA sequencing (RNA-seq) assisted prediction. The protein sequences from 7 species (*Pediculus humanus, Frankliniella occidentalis, Tribolium castaneum, Drosophila melanogaster, Apis mellifera, Caenorhabditis elegans*, and *Daphnia magna*) were fed to Maker for homology-based searches. The RNA-seq data were assembled using Trinity v2.11.0 (Trinity, RRID:SCR_013048) [48] software with the default parameters, and the output transcripts were set as mRNA evidence. BLAST v2.10.0 (NCBI BLAST, RRID:SCR_004870) [49] and Exonerate v2.58.3 (Exonerate, RRID:SCR_016088) [50] were used to search and polish the homologous sequences. The first-round output from the Maker analysis was collected and used to train gene models with SNAP v2006-07-28 (SNAP, RRID:SCR_007936) [51] and Augustus v3.3.3 (Augustus, RRID:SCR_008417) [52]. Gene models from both software programs were fed into Maker for the second-round run. Similarly, we ran a third round of gene model training and Maker prediction, after which we obtained the final version of the structural annotation results. Functional annotations were conducted on protein sequences using (i) DIAMOND BLASTP v2.0.14 [53] against the NCBI nr database; (ii) InterProScan v1.8.0_312 (InterProScan, RRID:SCR_005829) [54] on Gene Ontology (GO) terms, Signal peptides (SignalP), and InterPro annotations; and (iii) eggNOG-mapper v2.1.7 (eggNOG-mapper, RRID:SCR_021165) [55] with Clusters of Orthologous Genes (COG) category and KEGG pathways annotated.

### Orthology prediction and phylogenetic analyses

Insects from Hemiptera (*Acyrthosiphon pisum* and *Bemisia tabaci*), Thysanoptera (*F. occidentalis* and *Thrips palmi*), Psocodea (*L. brunnea, Columbicola columbae*, and *P. humunus*), and Holometabola (*D. melanogaster, Plutella xylostella*, and *T. castaneum*) were used in the orthology analysis with *Daphnia pulex* as the outgroup. Gene families including orthologous and paralogous gene families were detected by OrthoFinder v2.5.1 (OrthoFinder, RRID:SCR_017118) [56] using the default parameters. The protein sequences of all single-copy genes were aligned using MAFFT v7.475 (MAFFT, RRID:SCR_011811) [57] and concatenated into a data set. This data set was used to construct a phylogenetic tree using FastTree v2.1.10 (FastTree, RRID:SCR_015501) [58]. MCMCTREE from PAML package v4.9 (PAML, RRID:SCR_014932) [59] was used to date this phylogenetic tree. We retrieved the divergence time between (i) *D. melanogaster*and *P. xylostella* (243–317 million years ago [mya]) and (ii) *A. pisum* and *B. tabaci* (158–351 mya) from the TimeTree database [60].

### Gene family expansion, contraction andm annotation

The 11 species used in the previous section (*Orthology prediction and phylogenetic analyses*) were selected to identify gene family expansion and contraction. CAFE v4.2.1 (CAFE, RRID:SCR_005983) [61], which leverages a birth and death rate model estimated over the inferred phylogeny, was used to compare gene family cluster expansion and contraction (-p 0.01). The gene family clusters were then annotated by selecting the dominant function across all their genes using KinFin v1.0 [62].

For each gene family, we manually annotated 5 insecticide resistance-related gene families, ABC (ATP-binding cassette), EST, GST, P450, and UDP-glucuronosyl transferases (UGT); the heat shock protein (HSP) gene family; and 3 sensing-related gene families, CSP (chemosensory proteins), OBP (odorant–binding receptors), and OR (odourant receptors). The hidden Markov models (HMMs) of these gene families were downloaded from the Pfam database. The proteins of each gene family from *P. humanus, D. melanogaster*, and *Bactrocera dorsalis* were downloaded. The HMMs and proteins were fed as the input for BLASTP v2.10.0 (BLASTP, RRID:SCR_001010) and HMMER v3.1b2 (Hmmer, RRID:SCR_005305) to search for related genes. BITACORA v1.3 [63] was used to incorporate both results in protein mode with an e-value of 1e-5. Protein sequences of the annotated P450 and HSP genes were aligned using MUSCLE v3.8.1551 (MUSCLE, RRID:SCR_011812) [64]. Both alignments were used to construct neighbor-joining trees using TreeBeST v1.9.2 (TreeBeST, RRID:SCR_018173) with 1,000 rounds of bootstrap testing. The trees were annotated and viewed using FigTree v1.4.2 (FigTree, RRID:SCR_008515).

### Transcriptome analysis under phosphine fumigation/high temperature

We placed 40 adult females under phosphine (0.075 mg/L) for 2 hours as the insecticide treatment. High-temperature treatment employed 40 adult females, which were subjected to a temperature of 44°C for 2 hours. After both treatments, total RNA was immediately extracted. The treatment and control groups were replicated 4 times with 12 transcriptomes sequenced. The RNA extraction and sequencing processes followed the methods described earlier (*DNA extraction, RNA extraction, library construction, and sequencing*). After the quality control process, the sequencing data were mapped to the genome using HISAT2 v 2.2.1 (HISAT2, RRID:SCR_015530) [65] and quantified using FeatureCounts v2.0.1 (featureCounts, RRID:SCR_012919) [66]. Differentially expressed genes were analyzed using edgeR v3.32.1 (edgeR, RRID:SCR_012802) [67].

## Results

### Genome sequencing and assembly

**Figure 2.**
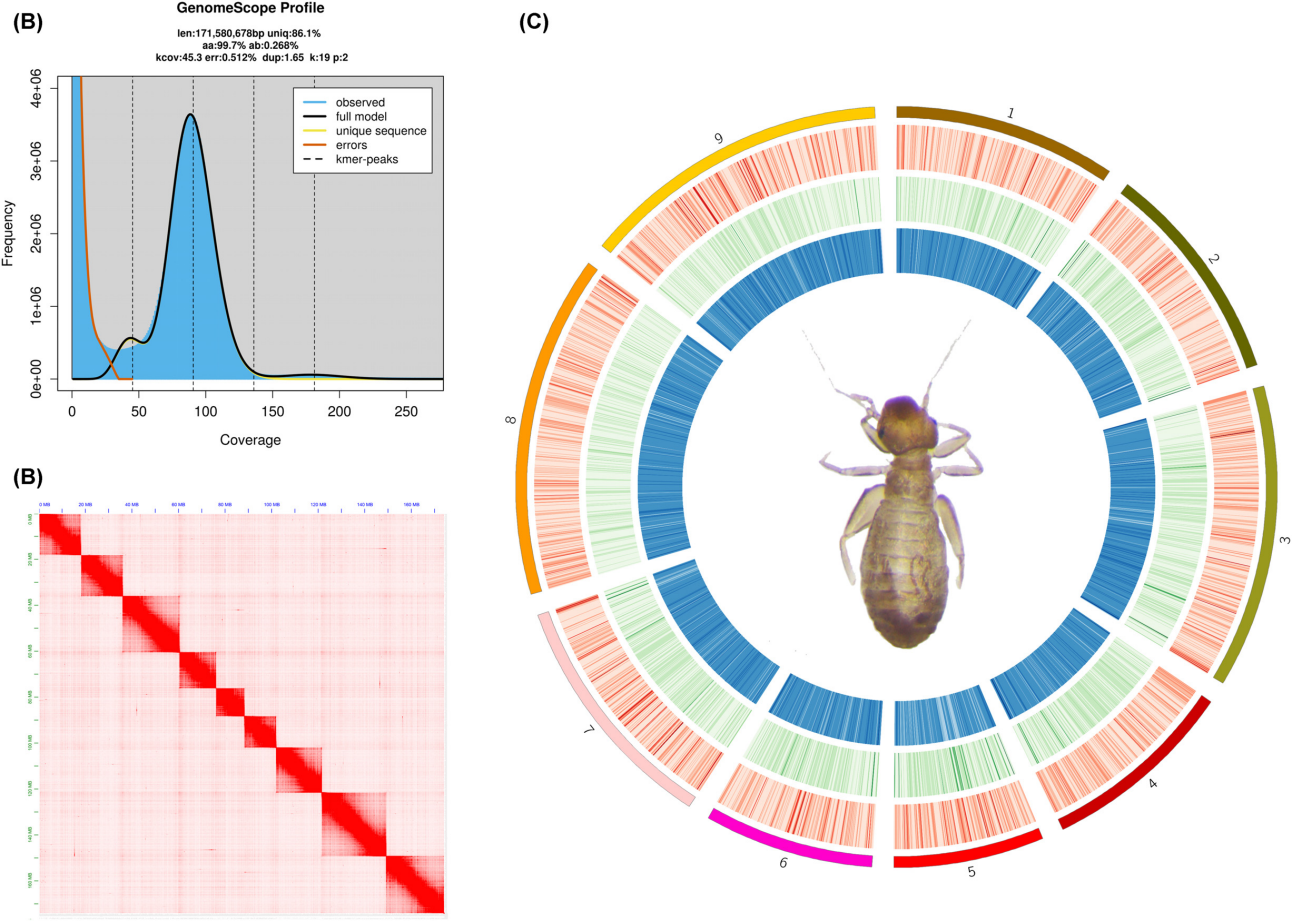
Genome survey, HiC and basic genomic features of *Liposcelis brunnea*. (A) GenomeScope estimation of genome size and heterogeneity using a k-mer of 19. (B) HiC interaction map produced by 3D-DNA. (C) Circos plot of the linkage groups. The outer 9 bars represented 9 linkage groups while the red, green, and blue heat maps represented gene counts, repeat sequences, and GC content, respectively, in 50-kb windows.

Altogether, 20.7 Gb of clean genome data (69,116,628 paired reads) were generated from the Illumina sequencing platform. The genome size was estimated to be 171.6 Mb with a heterogeneity of 0.268% (Fig. 2A). We obtained 52 Gb PacBio CLR data (2,733,343 subreads), which showed approximately 300-fold coverage with the subread N50 at 22.7 kb. After PacBio data correction, trimming, and assembly by Canu, a draft genome was generated, including 2,071 contigs with a total size of 283.8 Mb and a contig N50 of 800 kb. The genome size was about 110 Mb larger than the surveyed genome, indicating that some heterogeneous contigs existed in this draft genome. The result of the BUSCO analysis also indicated the presence of redundant sequences, including 98.8% complete genes (C), of which 65.2% were single-copy genes (S) and 33.6% were duplicated genes (D), 0.4% were partial genes (F), and 0.8% were missed genes (M). We noticed good completeness but a high percentage of duplicated genes, which could be the result of redundant contigs. Insect species often possess a high heterogeneity that requires redundancy purging after the initial genome assembly [68]. After polishing the draft genome using PacBio and the Illumina sequencing data, purge_dups was used to purge the redundancy and produced a purged genome including 278 contigs, 178.9 Mb in size, with a contig N50 of 1.78 Mb. The size of the purged genome is quite similar to our survey estimation and was subsequently used for the following HiC analysis.

Approximately 65.2 Gb of HiC data (217,199,354 read pairs) were produced and used to construct a chromosome-level genome assembly. After mapping the data to the purged genome, 162,444,941 unique read pairs were retrieved, including 150,305,169 valid interaction read pairs, which indicated good-quality HiC data (Fig. 2B). After manual checking, we obtained a genome assembly with the longest 9 linkage groups (LGs) covering 174.1 Mb (97.3% of genome bases). These LGs ranged from 12.1 to 27.6 Mb in length and reached a scaffold N50 of 19.7 Mb (Fig. 2C). The BUSCO evaluation result of the 9 LGs was C:98.9% [S:98.0%, D:0.9%], F:0.5%, M:0.6%. Compared with the only 2 genomes available in Psocodea (*P. humanus* and *C. columbae*), *L. brunnea* had a moderate genome size (Table 1) but had the largest contig N50, scaffold N50, and the best completeness evaluation, which indicated a high-quality genome.

**Table 1:** The genome features of *Liposcelis brunnea* and 2 parasitic lice

	Parasitic lice		Booklice
Genome features	*Pediculus humanus*	*Columbicola columbae*	*Liposcelis brunnea*
Genome size (MB)	110	208	174
Chromosomes	6	12	9
Methods	Capillary Platform	Nanopore + Illumina + HiC	PacBio + Illumina + HiC
Contig N50	—	511 kb	1.78 Mb
Scaffold N50	488 kb	17.6 Mb	19.7 Mb
Genes	10,773	13,362	15,543
Repetitive elements	7.3% (8.0 Mb)	9.7% (20.2 Mb)	15.9% (27.7 Mb)
BUSCO evaluation	95.9%	96.4%	97.2%

### Genome annotations

The structural annotation diagnosed 27,716,126 bp repeated sequences, constituting 15.92% of the *L. brunnea* genome. Retroelements and DNA transposons accounted for 3.81% and 1.24% of the genome, respectively. Of the retroelements, 2.61% of the genome sequence was identified as long interspersed elements, 1.18% as long terminal repeats, and 0.03% as short interspersed elements. There were also rolling circles (0.62%), satellites (0.04%), simple repeats (0.98%), low complexity (0.36%), and unclassified repeat sequences (8.87%). The content of repetitive elements typically correlates with genome size [69, 70], whereas exceptions exist in many cases partially because of the purging of heterogeneous contigs, or the nature of specific organisms [68,71]. Compared against *P. humanus* and *C. columbae* (Table 1), *L. brunnea* had the largest fraction of repetitive elements but with an intermediate genome size. The reduced size of transposable elements is considered common in lice and thus could be one reason for the reduction in genome size [31, 72]. Moreover, the reduction of certain gene families, such as those related to sensing, also accounts for the tightening of louse genomes [30].

After Maker gene annotation, 15,543 genes were annotated in the genome of *L. brunnea*. The BUSCO result of this gene set was C:97.2% [S:95.8%, D:1.4%], F:1.2%, and M:1.6%, indicating good-quality structural annotation. Among all 15,543 genes, 12,157 genes were annotated by the nr database; 10,724 genes were annotated by InterProScan, with confirmed GO, SignalP, and InterPro terms; and 10,097 genes were annotated by eggNOG-mapper, together with the COGs and KEGGs. *L. brunnea* had 4,770 and 2,181 more genes than *P. humanus* and *C. columbae*, respectively. Regardless, compared with the other 7 insect genomes, *P. humanus* and *C. columbae* also had the smallest gene numbers, indicating a large number of gene reductions in parasitic lice.

### Gene orthology analysis and phylogeny reconstruction

**Figure 3.**
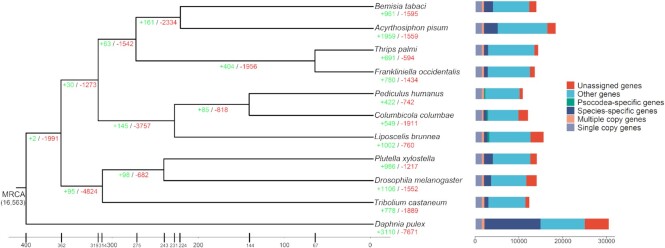
Gene family evolution among *Liposcelis brunnea* and other insects. Phylogenetic trees inferred from concatenated single-copy genes using FastTree. MCMCTREE was used for molecular dating. The single-copy genes, multiple-copy genes, species-specific genes, and clade-specific genes were analyzed based on the results from OrthoFinder. Gene family expansion (green) and contraction (red) were analyzed using CAFE.

In total, 16,563 gene families were identified, of which 1,448 were single-copy genes in the OrthoFinder analysis (Fig. 3A). For *L. brunnea*, we assigned 12,530 genes to 9,144 gene families with 813 species-specific genes. For these arthropods, the unique genes ranged from 47 to 3,117, representing their specific evolutionary pathways, which will be explained in detail using gene family analysis.

Based on the phylogenetic reconstruction using single-copy genes, all species of Paraneoptera (Psocodea, Hemiptera, and Thysanoptera) formed a clade, whereas other insects clustered together. However, several recent studies [26] reported that Psocodea might cluster with Holometabola insects but not with Hemiptera + Thysanoptera insects, which could be caused by unbalanced sampling and the different gene data sets used for phylogenetic inference. Our results thus indicated a closer relationship of the Paraneoptera clades. The MCMCTREE result indicated a divergence time between booklice and parasitic lice at ∼231 mya, which is similar to the results of several previous studies [25].

### Gene family expansion and contraction

We first detected how the gene family evolved in Psocodea using CAFE, which might account for the formation of parasitism (Fig. 3). For the MRCA of booklouse (*L. brunnea*) and parasitic lice (*P. humanus* and *C. columbae*), 145 gene families expanded, whereas 3,757 contracted. The large number of contracted gene families indicates potential biological functional loss [30]. Indeed, gene families including P450s, G protein-coupled receptors (GPCRs), OR, gustatory receptor (Gr), and CSP were contracted in both *P. humanus* and *C. columbae* (Supplementary Table S1). Similar gene family (GPCRs and P450s) contraction was observed in the MRCA of booklice and parasitic lice, whereas there was a gene family expansion of Gr genes and no change in OR and CSP genes. These results indicate that the sense-related gene family changed mainly in the parasitic lice but not in the booklice. Conversely, the gene families of GPCR, OR, CSP, and P450 expanded significantly in *L. brunnea*, which might be explained by the requirements of a free-living lifestyle and adaptation to environmental change [73,74].

The BITOCORA analyses (Table 2) confirmed the Kinfin results with the 3 sensing-related gene families (CSP, OR, and OBP) contracted in parasitic lice and expanded in *L. brunnea*. Surprisingly, although parasitic lice and booklice live in different temperature conditions, they still have similar HSP gene numbers (∼40). Moreover, we found that all insecticide resistance-related gene families (ABC, EST, GST, UGT, and P450) kept their numbers in *L. brunnea* but were contracted in the 2 parasitic lice, indicating less environmental challenge to the latter clade [75, 76].

**Table 2:** Statistics on detoxification, heatshock protein (HSP), and sensing-related genes across Psocodea insects and other insects

	Psocodea				Hemiptera				Thysanoptera			Diptera
Gene family	*Pediculus humunus*	*Columbicola columbae*	*Liposcelis brunnea*		*Acyrthosiphon pisum*	*Bemisia tabaci*	*Nilaparvata lugens*		*Frankliniella occidentalis*	*Thrips palmi*		*Drosophila melanogaster*
ABC	43	47	**66**		107	53	80		65	57		57
EST	26	29	**69**		45	49	81		66	78		42
GST	24	18	**44**		34	32	26		33	36		54
UGT	4	3	**19**		60	80	20		26	18		35
P450	43	44	**125**		79	141	88		95	115		92
HSP	41	39	**42**		48	44	71		71	63		58
CSP	6	8	**9**		10	19	17		11	11		4
OBP	3	5	**37**		18	8	23		16	24		47
OR	10	9	**29**		17	10	31		13	15		67

### P450 genes in phosphine resistance

**Figure 4.**
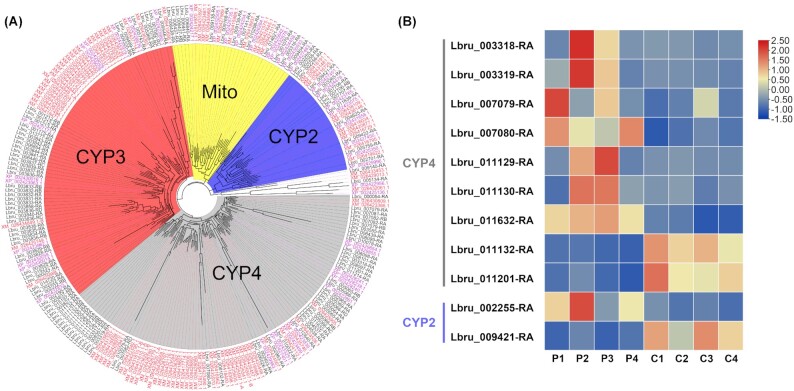
Phylogenetic tree of P450 gene family and significantly expressed genes. (A) The P450 genes of *L. brunnea* (in black), *P. humanus* (in purple), *and D. melanogaster* (in red) from BITACORA analysis were used to construct a neighbor-joining tree where 4 subfamilies separated obviously. (B) Four treatment (P1–P4) and 4 control (C1–C4) groups were analyzed. The subfamilies of 11 differentially expressed P450 genes are listed. The expression data were normalized for each gene.

Among all insecticide resistance-related gene families, we noticed that the P450 gene family was very large, with 125 P450 genes (Table 2). Regardless, *L. brunnea* has a large P450 gene family compared with all other closely related species. Four P450 subfamilies (CYP2, CYP3, CYP4, and Mito) of *L. brunnea* included respectively 13, 44, 50, and 16 genes, whereas *F. occidentalis* had 10, 29, 43, and 10 genes and *P. humunus* had 7, 12, 11, and 10 genes for each subfamily (Fig. 4A). Compared with the parasitic lice, all 4 subfamilies of *L. brunnea* expanded significantly. The CYP4 subfamily had the largest number of genes, which could be the potential reason for high insecticide resistance. Similar CYP4 subfamily expansion was observed in *Thrips palmi*, which partially accounted for its high insecticide resistance [77].

We then analyzed how P450 genes reacted to phosphine fumigation. After RNA-seq analyses, under the criteria of *P* < 0.05 and mRNA expression fold change >2, we found 11 differentially expressed genes (DEGs) from the P450 gene family (Fig. 4B, Supplementary Table S2), distributed in the CYP4 (9 genes) and CYP2 (2 genes) subfamilies. Two DEGs were from the CYP2 subfamily with one upregulated and the other downregulated. As predicted, most DEGs were from the CYP4 subfamily, 7 of which were upregulated, suggesting that the largest P450 subfamily (CYP4) had the most important biological function with regard to phosphine resistance. Our results indicated that the high fumigant resistance in booklice species might originate from the expansion of the P450 gene family, particularly its CYP4 subfamily.

### HSP genes in heat tolerance

Based on the fact that booklice favor high temperatures and HSP genes function during heat treatment across many species [78], we hypothesized that the free-living booklice possess an expanded HSP gene family. However, our gene family analyses proved that the HSP gene family of *L. brunnea* had a small number of genes across all species (Table 2). All 3 lice had approximately 40 P450 genes, indicating a similar evolutionary pathway in Psocodea (Psocoptera + Phthiraptera). There could be two reasons for the conservation of HSP genes: (i) as an epibiont, parasitic lice still suffer fluctuating temperatures under various host activities, or (ii) HSP genes are key components of other necessary biological functions (i.e., insect sleep) [79] and thus are not influenced only by temperature conditions.

**Figure 5.**
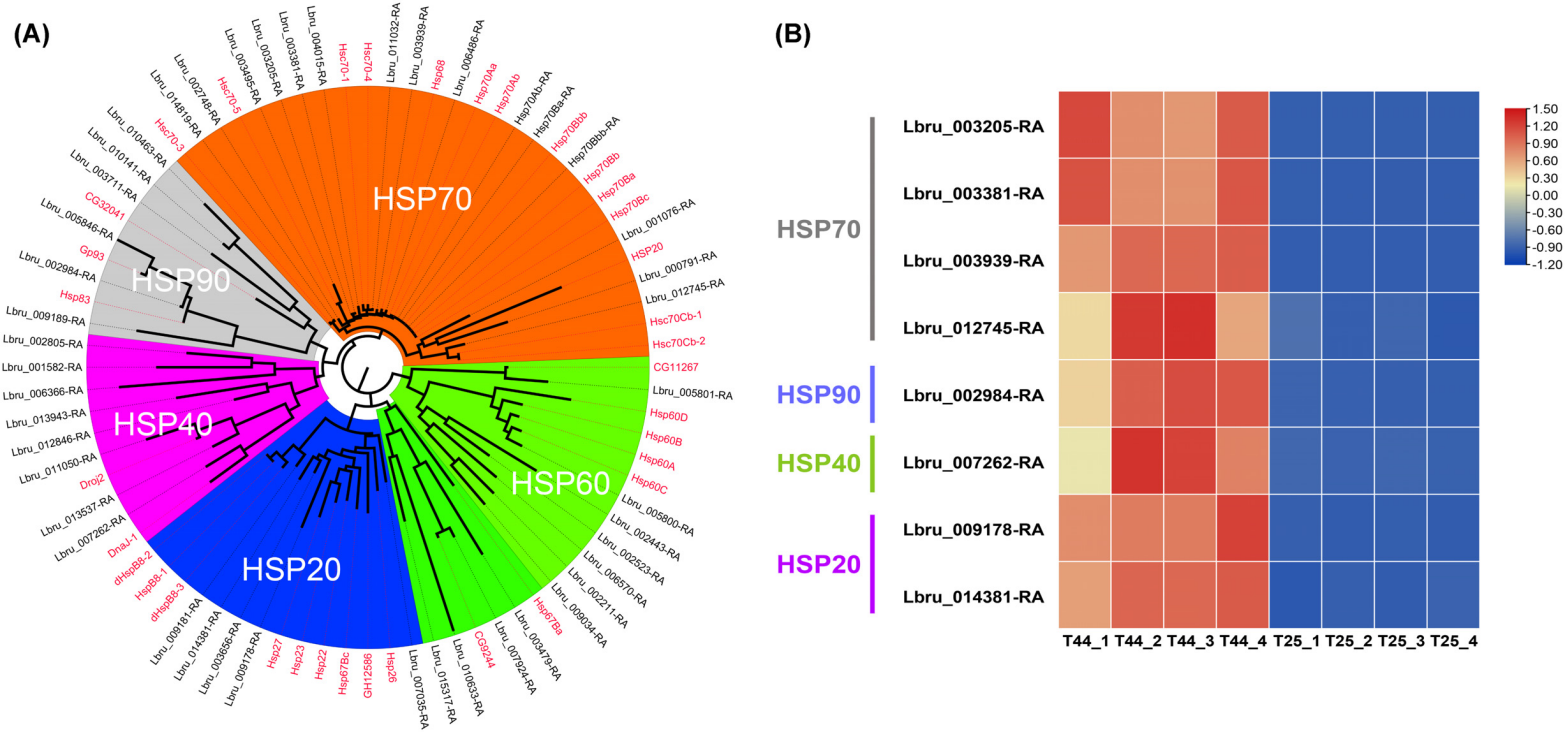
Phylogenetic tree of HSP gene family and significantly expressed genes. (A) The HSP genes of *L. brunnea* (in black) and *D. melanogaster* (in red) from BITACORA analysis were used to construct a neighbor-joining tree where 5 subfamilies separated obviously. (B) Four treatment (T44_1–T44_4) and 4 control (T25_1–T25_4) groups were analyzed. The subfamilies of 8 differentially expressed HSP genes are listed. The expression data were normalized for each gene.

Five HSP subfamilies were identified in *L. brunnea* (Fig. 5A), including HSP20 (5), HSP40 (8), HSP60 (11), HSP70 (15), and HSP90 (6). After RNA-seq analysis, we found that 8 HSP genes from 4 subfamilies were upregulated significantly, indicating the importance of HSP genes for heat adaptation in booklice (Fig. 5B). HSP genes have been proven to be key in temperature adaptation in insects [80]. Our findings confirm these results and provide further evidence for how psocids have adapted to this important ecological aspect.

## Conclusions

We report a high-quality genome assembly of *L. brunnea*, the type species in the genus *Liposcelis*. This is the first genome from the order Psocoptera uncovered. The genome of *L. brunnea* has a contig N50 of 1.78 Mb and is distributed into 9 LGs. The lice clade, including booklice, barklice and parasitic lice, diversified for approximately 231 million years with sensing- and insecticide resistance-related gene families contracted in the latter clade. We found that P450 genes, particularly those from the CYP4 subfamily, affect phosphine fumigation and thus are key potential targets for genetic-based pest control methods. Approximately one-quarter of the HSP genes were upregulated under heat treatment, indicating their importance in temperature adaptation. Overall, our study provides valuable data and insights into lice evolution and environmental stress adaptation.

## Additional Files

**Supplementary Table S1**. Annotation of clade-specific expansion/contraction gene families from KinFin analysis.

**Supplementary Table S2**. Differential expressed genes in P450 gene family during phosphine fumigation.

giac062_GIGA-D-22-00060_Original_Submission

giac062_GIGA-D-22-00060_Revision_1

giac062_Response_to_Reviewer_Comments_Original_Submission

giac062_Reviewer_1_Report_Original_Submission Omaththage Perera -- 4/20/2022 Reviewed

giac062_Reviewer_1_Report_Revision_1 Omaththage Perera -- 5/5/2022 Reviewed

giac062_Reviewer_2_Report_Original_Submission Shu-Jun Wei -- 4/23/2022 Reviewed

giac062_Reviewer_2_Report_Revision_1 Shu-Jun Wei -- 5/5/2022 Reviewed

giac062_Supplemental_Tables

## Abbreviations

BUSCO: Benchmarking Universal Single-Copy Orthologs; Gb: Gigabase; Mb: Megabase; kb: Kilobase;bp: base pair; RNA-seq: RNA sequencing; mRNA: messenger RNA; NCBI: The National Center for Biotechnology Information; nr: non-redundant; KEGG: Kyoto Encyclopedia of Genes and Genomes.

## Data Accessibility

Illumina DNA/RNA sequencing data, PacBio sequel II genome sequencing, and HiC data were uploaded at NCBI SRA under BioProject: PRJNA772023. The genome assembly is under NCBI WGS Accession: JAJEOV000000000. All other supporting data and materials are available in the *GigaScience* GigaDB database [81].

## Competing Interests

The authors declare that the research described herein was conducted in the absence of any commercial or financial relationships that could be construed as a potential competing interests.

## Funding

This work was supported by the Key Research Program of International Collaboration between China and Czech Republic (2018YFE0108700) to Z.L. and the China Agriculture Research System of MOF and MARA to Z.L.

## Authors’ Contributions

S.F. and Z.L. conceived the project and wrote the manuscript. G.O., V.S., and Z.L. collected and identified the samples. S.F. performed the analyses. S.F. and W.D. performed the experiments. All authors read and approved the final manuscript.
