# Supplementary material for: A chromosome-level genome of the booklouse, Liposcelis brunnea, provides insight into louse evolution and environmental stress adaptation
Source: Gigascience. 2022 Jul 19;11:giac062. doi: 10.1093/gigascience/giac062 (PMC9295366; doi:10.1093/gigascience/giac062)
Supplement: giac062_GIGA-D-22-00060_Original_Submission [file giac062_giga-d-22-00060_original_submission.pdf]

GigaScience

A chromosome-level genome of the booklouse, Liposcelis brunnea provides insight into lice evolution and environmental stress adaptation

--Manuscript Draft--

**Manuscript Number:**	GIGA-D-22-00060
**Full Title:**	A chromosome-level genome of the booklouse, Liposcelis brunnea provides insight into lice evolution and environmental stress adaptation
**Article Type:**	Data Note
**Funding Information:**	Key Research Program of International Collaboration between China and Czech Republic (2018YFE0108700) — Professor Zhihong Li<br>China Agriculture Research System of MOF and MARA — Professor Zhihong Li
**Abstract:**	Background Booklice (psocids) in the genus Liposcelis (Psocoptera: Liposcelididae), are a group of important storage pests, found in libraries, grain storages and food processing facilities. Booklice are able to live under heat treatment and usually possess high resistance to common fumigant insecticide, hence a threat to storage security worldwide.Results We assembled the genome of the booklouse, L. brunnea, the first genome reported in Psocoptera, using PacBio long-read sequencing, Illumina sequencing and HiC methods. After assembly, polishing, haplotypes purging and HiC scaffolding, we got nine linkage groups (174.1 Mb totally), ranging from 12.1 Mb to 27.6 Mb (N50: 19.7 Mb), with the BUSCO completeness at 98.9%. Totally 15,543 genes were predicted by the MAKER pipeline. Gene family analyses indicated the sensing-related gene families (OBP, OR) and the resistance-related gene family (ABC, EST, GST, UGT, P450) expanded significantly compared to its closest relatives (two parasitic lice). Based on transcriptomic analysis, we found the CYP4 subfamily from P450 gene family functioned during phosphine fumigation; HSP genes especially those from the HSP70 subfamily upregulated significantly in countering high temperatures.Conclusions We present a high-quality genome assembly of L. brunnea, filling in the blanks in this clade. Furthermore, our results provide new insights into lice evolution and the strategy on how insect adapts to environmental stresses.
**Corresponding Author:**	Shiqian Feng<br>China Agricultural University<br>Beijing, CHINA
**Corresponding Author Secondary Information:**	
**Corresponding Author's Institution:**	China Agricultural University
**Corresponding Author's Secondary Institution:**	
**First Author:**	Shiqian Feng
**First Author Secondary Information:**	
**Order of Authors:**	Shiqian Feng<br>George Opit<br>Wenxin Deng<br>Vaclav Stejskal<br>Zhihong Li
**Order of Authors Secondary Information:**	
**Additional Information:**	
**Question**	**Response**

Powered by Editorial Manager® and ProduXion Manager® from Aries Systems Corporation

Are you submitting this manuscript to a special series or article collection?	No
**Experimental design and statistics**<br><br>Full details of the experimental design and statistical methods used should be given in the Methods section, as detailed in our Minimum Standards Reporting Checklist. Information essential to interpreting the data presented should be made available in the figure legends.<br><br>Have you included all the information requested in your manuscript?	Yes
**Resources**<br><br>A description of all resources used, including antibodies, cell lines, animals and software tools, with enough information to allow them to be uniquely identified, should be included in the Methods section. Authors are strongly encouraged to cite Research Resource Identifiers (RRIDs) for antibodies, model organisms and tools, where possible.<br><br>Have you included the information requested as detailed in our Minimum Standards Reporting Checklist?	Yes
**Availability of data and materials**<br><br>All datasets and code on which the conclusions of the paper rely must be either included in your submission or deposited in publicly available repositories (where available and ethically appropriate), referencing such data using a unique identifier in the references and in the "Availability of Data and Materials" section of your manuscript.<br><br>Have you have met the above requirement as detailed in our Minimum	Yes

Standards Reporting Checklist?	

Powered by Editorial Manager® and ProduXion Manager® from Aries Systems Corporation

Manuscript
Click here to access/download;Manuscript;Main_Manuscript.docx

A chromosome-level genome of the booklouse, *Liposcelis brunnea* provides insight into lice evolution and environmental stress adaptation

Shiqian Feng[1,2], George Opit[3], Wenxin Deng[1,2], Vaclav Stejskal[4,5], Zhihong Li[1,2,*]

1. Department of Plant Biosecurity, College of Plant Protection, China Agricultural University, Beijing 100193, China

2. Key Laboratory of Surveillance and Management for Plant Quarantine Pests, Ministry of Agriculture and Rural Affairs, Beijing 100193, China

3. Department of Entomology and Plant Pathology, Oklahoma State University, Oklahoma 74078, USA

4. Crop Research Institute, Drnovská 507, 161 06 Prague 6, Czech Republic

5. Czech University of Life Sciences, Faculty of Agrobiology, Food and Natural Resources, Kamycka 129, 165 00 Prague, Czech Republic

* Corresponding author: lizh@cau.edu.cn

Abstract

Background Booklice (psocids) in the genus *Liposcelis* (Psocoptera: Liposcelididae), are a group of important storage pests, found in libraries, grain storages and food processing facilities. Booklice are able to live under heat treatment and usually possess high resistance to common fumigant insecticide, hence a threat to storage security worldwide.

Results We assembled the genome of the booklouse, *L. brunnea*, the first genome reported in Psocoptera, using PacBio long-read sequencing, Illumina sequencing and HiC methods. After assembly, polishing, haplotypes purging and HiC scaffolding, we got nine linkage groups (174.1 Mb totally), ranging from 12.1 Mb to 27.6 Mb (N50: 19.7 Mb), with the BUSCO completeness at 98.9%. Totally 15,543 genes were predicted by the MAKER pipeline. Gene family analyses indicated the sensing-related gene families (OBP, OR) and the resistance-related gene family (ABC, EST, GST, UGT, P450) expanded significantly compared to its closest relatives (two parasitic lice). Based on transcriptomic analysis, we found the CYP4 subfamily from P450 gene family functioned during phosphine fumigation; HSP genes especially those from the HSP70 subfamily upregulated significantly in countering high temperatures.

Conclusions We present a high-quality genome assembly of *L. brunnea*, filling in the blanks in this clade. Furthermore, our results provide new insights into lice evolution and the strategy on how insect adapts to environmental stresses.

Keywords: Booklice, *Liposcelis brunnea*, genome assembly, lice evolution, insecticide resistance

1. Introduction

Psocids are stored product arthropods that are of increasing economic importance as pests of seeds, raw agricultural materials, food and feed [1–4]. Booklice in the genus *Lipocelis* are the most important clade across psocids because of their worldwide distribution and high resistance to insecticides and fumigants [5]. More generally, booklice were known as minute, pale insects found scuttling across books or stacks of papers [6]. Booklice infestations are usually a result of poor storage conditions associated with high moisture, which negatively influences the commodity [7]. Psocid feeding can cause 5-10% weight loss in agricultural commodities [8,9]. They can also have negative impacts on human health through production of allergens [10,11] or transmitting parasites [12].

Contact insecticides and fumigants are used for managing booklice; however, they are tolerant or can quickly develop resistance compared to other stored products pests [2]. For example, deltamethrin, carbaryl, and methoprene which which could control beetles and moths are not effective against booklice [13,14]. Booklice have resistance to the bacterium-derived spinosad, imidacloprid and diatomaceous earth [15,16]. Phosphine fumigation is the most popular method for managing storage pests [17]. High levels of resistance to phosphine exist in booklice, especially during their egg stages, which significantly increases both the economic cost of treatments and environmental pollution [18,19]. Esterases (EST, Wei et al. 2020)[20], glutathione S-transferase (GST, Wu et al. 2009) [21], Cytochrome P450 monooxygenases (P450, Wei et al. 2014) [22] have been shown to be associated with high resistances in *Liposcelis* species. Recently, using transcriptomics data, Wei et al. (2020) proposed the P450 and GST genes might account for the high metabolic resistance found in booklice. However, the mRNA level of data reflects that those genes with high expression and

under certain treatments can prejudice gene family evaluation [23]. As a result, it is necessary to develop a nuclear genome of booklice species for resistance related analyses.

Booklice are the phylogenetic sister group to parasitic lice and they have been considered as a key taxon in uncovering the origins and evolution of parasitic lice [24–26]. The habits of *Liposcelis* species are similar to those of parasitic lice, for example, the fact that they are found in nests of birds and mammals [27–29]. Therefore, identifying the shared features of booklice and parasitic lice, especially their genome features, might provide unique insight into the origin of parasitism. To date, two parasitic genomes have been published while no booklouse genome has been sequenced [30,31].

Here we present a high-quality genome assembly of *L. brunnea* (Fig. 1). It is the first chromosome-level genome assembly in Psocoptera and was assembled based on Pacbio sequencing, illumina and chromatin conformation capture (HiC) technology. Comparative genomics and transcriptomic analyses provided new clues on the evolution of lice, especially how the booklice adapted to high temperature and insecticides.

2. Methods

2.1 Samples collection

Samples of *L. brunnea* were collected in Oklahoma State, United States. They were put in jars and fed on a mix of wheat flour: yeast: whole milk powder = 10:1:1. The rearing jars were put into incubators under a dark environment with the temperature and relative humidity setting to 25℃ and 75%, respectively.

2.2 DNA extraction, RNA extraction, library construction, and sequencing

Genomic DNA of 500 female adults was extracted using Promega Genomic DNA Purification Kit (A1125). After examination of the quality of isolated DNA, the library of 20 kb was constructed using a SMRTbell Express Template Prep Kit 2.0 (Pacific Biosciences, CA, USA). The construction includes DNA shearing, damage repair, end repair, hairpin adapter ligation, and purification of the library. After a quality control test, a SMRTbell library was obtained. The library was sequenced using a single 8 M SMRT Cell on the PacBio Sequel II platform (Pacific Biosciences, CA, USA) (PacBio Sequel II System). For genome survey and assembly polishing, we extracted genomic DNA from 40 female adults and constructed an Illumina sequencing library according to the manufacturer's introductions (Illumina), which was then sequenced on the Illumina Novaseq 6000 platform with paired-end 150 bp mode (insert size 350 bp) for about 20 GB data. For genome annotation, we extracted total RNA from 40 female adults using the Tiangen RNA extraction kit. After transferring mRNA into cDNA, another Illumina library was constructed and sequenced with the same parameters for about 6 GB data. The sequencing processes were conducted on the platforms of Berry Genomics Company.

2.3 HiC sequencing

Approximately 500 female adults were collected for HiC experiments and sequencing. The library was constructed through the following steps: crosslinking the crashed samples with formaldehyde, digesting DNA with MboI enzyme, filling ends and marks with biotin, ligating the resulting blunt-end fragments, purification and random shearing DNA into 300-500 bp fragments. After library construction following the manufacturer's introductions (Illumina), sequencing was performed on the Illumina Novaseq 6000 platform with 150 bp

pair-ended mode for about 60 GB data. The experiments and sequencing were performed by Annoroad Gene Technology.

2.4 Genome survey, assembly, quality assessment

Using the Illumina sequencing reads, we counted the 19-mers with Jellyfish v2.2.10 [32] and evaluated the genome features using GenomeScope v2.0 [33]. The Pacbio CLR data was processed using Canu v2.1.1 [34] following the correction (-correct), trimming (-trim) and assembly (-assemble), with the parameters: minReadLength=2000, minOverlapLength=500, corOutCoverage=120, corMinCoverage=2, correctedErrorRate=0.035. Pacbio sequencing data and Illumina sequencing data were both leveraged to polish the draft genome. The Pacbio sequencing data was mapped to the draft genome using pbmm2 v1.4.0, after which gcpp v1.9.0 with arrow algorithm was used for assembly polish. The pbmm2 and gcpp software were from Pacific Biosciences official toolkits (https://github.com/PacificBiosciences). We then mapped the Illumina sequencing data to the gcpp-polished assembly using BWA v0.7.17 [35], and Pilon v1.23 [36] was used to polish for the second round. Because we set "correctedErrorRate" to a very low level in the Canu assembly step, the heterogeneous contigs were separated, producing redundant contigs. So after assembly polishing, purge_dups v1.2.5 [37] was used for redundancy purge. The filtered Hi-C reads were aligned to the polished genome by BWA v0.7.17 which was integrated into Juicer [38]. Only uniquely mapped and valid paired-end reads were used for assembly by 3D-DNA v180114 [39]. Juicebox [40] was used to manually order the scaffolds to get the final chromosome assembly. BUSCO v5.1.3 [41] was used to assess the completeness of the final genome assembly with arthropoda_odb10 database.

2.5 Genome annotation

RepeatModeler v2.0.1 [42] was used to build a custom *de novo* repeat library, based on which RepeatMasker v4.1.0 [43] was used to detect the repetitive elements. Genome structural annotation was conducted using the Maker pipeline v3.01.03 [44] with *ab initio* prediction, homology-based prediction and RNA-Seq assisted prediction. The protein sequences from seven species (*Pediculus humanus*, *Frankliniella occidentalis*, *Tribolium castaneum*, *Drosophila melanogaster*, *Apis mellifera*, *Caenorhabditis elegans*, *daphnia magna*) were fed to Maker for homology-based searches. The RNA-seq data was assembled using Trinity v2.11.0 [45] software with the default parameters and the output transcripts were set as mRNA evidence. BLAST v2.10.0 [46] and Exonerate v2.58.3 [47] were used to search and polish the homologous sequences. The first round output from Maker analysis was collected and used to train gene models by SNAP v2006-07-28 [48] and Augustus v3.3.3 [49], Gene models from which was fed into Maker for the second round run. Likewise, we ran a third round of gene model training and Maker prediction, after which we got the final version of structural annotation results. Functional annotations were conducted on protein sequences by 1) DIAMOND BLASTP [50] against NCBI nr database; 2) InterProScan [51] on gene ontology (GO) terms, Signal peptides (SignalP) and InterPro annotations ; 3) eggNOG-mapper [52], with COG category and KEGG pathways annotated.

2.6 Orthology prediction and phylogenetic analyses

Insects from Hemiptera (*Acyrthosiphon pisum*, *Bemisia tabaci*), Thysanoptera (*Frankliniella occidentalis*, *Thrips palmi*), Psocodea (*L. brunnea*, *Columbicola columbae*, *Pediculus humunus*) and Holometabola (*Drosophila melanogaster*, *Plutella xylostella*, *Tribolium castaneum*) were used in orthology analysis with *Daphnia pulex* as the outgroup.

Gene families including orthologous and paralogous gene families were detected by OrthoFinder [53] with the default parameters. The protein sequences of all single copy genes were aligned using MAFFT [54] and concatenated into a dataset. This dataset was used to construct a phylogenetic tree using Fasttree. MCMCTREE from PAML package v4.9 [55] was used to date this phylogenetic tree. We retrieved the divergence time between 1) *Drosophila melanogaster* and *Plutella xylostella* (243-317 MYA, million years ago), 2) *Acyrthosiphon pisum*, *Bemisia tabaci* (158-351 MYA) from TimeTree database (Hedges et al. 2006).

2.7 Gene family expansion, contraction and annotation

To identify gene family expansion and contraction in Psocodea, protein sequences from 11 species in Orthology analysis were selected with their phylogeny known. CAFE v4.2.1 [56], which leverages a birth and death rate model estimated over the inferred phylogeny, was used to compare gene family cluster expansion and contraction (-p 0.01). The gene family clusters were then annotated by selecting the dominant function across all their genes using KinFin v1.0 [57]. Moreover, we conducted protein domain searches using HMMER v3.1b2 [58] for the non-redundant protein sequences of *L. brunnea*, *P. humanus, D. melanogaster*. The e-value was set to 1e-10 and the domains of *L. brunnea* were compared against *P. humanus*, *D. melanogaster*, respectively. Domain counting and comparison was conducted using a custom script. The figures were plotted using the *ggplot2* package [59] in R v4.0.4 [60].

For each gene family, we manually annotated five gene resistance related families, ABC (ATP-binding cassette), EST, GST, P450, UGT (UDP glucuronosyltransferases); the HSP (heat shock protein) gene family; three sensing related gene families, CSP (chemosensory

proteins), OBP (odorant‑binding receptors), OR (odorant receptors). The hidden Markov models (HMMs) of these gene families were downloaded from the Pfam database. The proteins of each gene family from *P. humanus, D. melanogaster* and *Bactrocera dorsalis* were downloaded. The HMMs and proteins were fed as the input of BLASTP v2.10.0 and HMMER v3.1b2 to search for related genes. BITACORA v1.3 [61] was used to incorporate both results under protein mode and an e-value of 1e-5. Protein sequences of the annotated P450 and HSP genes were aligned using MUSCLE v3.8.1551 [62]. We then constructed neighbor-joining trees using TreeBeST v1.9.2 (https://github.com/Ensembl/treebest) with 1,000 rounds of bootstrap test. The trees were annotated and viewed using FigTree v1.4.2 (http://tree.bio.ed.ac.uk/software/figtree/).

2.8 Transcriptome analysis under phosphine fumigation/high temperature

We treated 40 female adults under phosphine (50 ppm)/44℃ and extracted the transcriptome after two hours' treatment. The treatment and control groups replicated four times with totally 12 transcriptomes sequenced. The RNA extraction, sequencing processes followed section *2.2 DNA extraction, RNA extraction, library construction, and sequencing*. After quality control process, the sequencing data was mapped to the genome using HISAT2 [63] and quantified using FeatureCounts [64]. Differential expressed genes were analysed using edgeR [65].

3. Results

3.1 Genome sequencing and assembly

Altogether, 20.7 Gb of clean genome data (69,116,628 paired reads) was generated from Illumina sequencing platform. The genome size was estimated by GenomeScope to be 171.6

Mb with the heterogeneity of 0.268% (Fig. 2A). We obtained 52 Gb Pacbio CLR data (2,733,343 subreads) which was about 300-fold coverage with the subreads N50 at 22.7 kb. After Pacbio data correction, trimming and assembly by Canu, a draft genome was generated, including 2,071 contigs with a total size of 283.8 Mb and contig N50 at 800 kb. The genome size is about 110 Mb bigger than the surveyed one, indicating some heterogeneous contigs existed in this draft genome. The result of BUSCO analysis also indicated the presence of redundant sequences. The BUSCO result is 98.8% complete genes (C) including [65.2% single copy genes (S) and 33.6% duplicated genes (D)], 0.4% genes are partial (F) and 0.8% genes missed (M). We noticed a good completeness but a high percentage of duplicated genes, which could be the results of redundant contigs. Insect species often possess a high heterogeneity that requires redundancy purging after the initial genome assembly [66]. After polishing the draft genome using Pacbio and Illumina sequencing data, purge_dups was used to purge the redundancy and produced a purged genome including 278 contigs, 178.9 Mb in size with the contig N50 of 1.78 Mb. The size of the purged genome is quite similar to our survey estimation and was subsequently used for the following HiC analysis.

About 65.2 Gb HiC data (217,199,354 read pairs) was produced and used to construct a chromosome-level genome assembly. After mapping the data to the purged genome, 162,444,941 unique read pairs were retrieved including 150,305,169 valid interaction read pairs, which indicated a good quality of HiC data (Fig. 2B). After manually checking, we obtained a genome assembly with the longest 9 linkage groups (LGs) covering 174.1 Mb (97.3% genome bases). These LGs ranged from 12.1 Mb to 27.6 Mb in lengths and reached a scaffold N50 of 19.7 Mb (Fig. 2C). The BUSCO evaluation result of nine LGs is C:98.9% [S:98.0%, D:0.9%], F:0.5%, M:0.6%. Compared to the only two genomes available in Psocodea (*P. humanus* and *C. columbae*), *L. brunnea* had a mediate genome size (Table 1),

but with the biggest contig N50, scaffold N50 and the best completeness evaluation, which indicated a high-quality genome.

3.2 Genome annotations

The structural annotation diagnosed 27,716,126 bp repeated sequences, constituting 15.92% of the *L. brunnea* genome. Retroelements and DNA transposons accounted for 3.81% and 1.24% of the genome, respectively. For retroelements, 2.61% genome sequence was identified as long interspersed elements (LINEs), 1.18% as long terminal repeats (LTRs), 0.03% as short interspersed elements (SINEs). There were also rolling-circles (0.62%), satellites (0.04%), simple repeats (0.98%), low complexity (0.36%) and unclassified repeat sequences (8.87%). The content of repetitive elements usually correlated with the genome size [67,68], whereas exceptions exist in many cases partially because of the purging of heterogeneous contigs, or the nature of specific organisms [66,69]. Compared against *P. humanus* and *C. columbae* (Table 1), *L. brunnea* had the biggest fraction of repetitive elements however with the intermediate level of genome size. The reduced size of transposable elements were considered to be common in lice, thus could be one reason for the genome size reduction [31,70]. Moreover, the reduction of certain gene families, such as those related to sensing, also accounted for the tightening of lice genomes [30].

After Maker gene annotation, in total 15,543 genes were annotated in the genome of *L. brunnea*. The BUSCO result of this gene set is C:97.2% [S:95.8%, D:1.4%], F:1.2%, M:1.6%, indicating a good quality of structural annotation. Among all 15,543 genes, 12,157 genes were annotated by nr database; 10,724 genes were annotated by Interproscan, with GO terms, SignalP and InterPro terms confirmed; 10,097 genes were annotated by eggNOG-mapper, together with the COGs, KEGGs. *L. brunnea* had 4,770 and 2,181 more genes than *P.*

humanus and *C. columbae*. Nonetheless, Compared to the other seven insect genomes, *P. humanus* and *C. columbae* also had the smallest gene numbers, indicating a large number of gene reductions in parasitic lice.

3.3 Gene orthology analysis and phylogeny reconstruction

In total 16,563 gene families were identified, with 1,448 of them as single copy genes in Orthofinder analysis (Fig. 3A). For *L. brunnea*, we assigned 12,530 genes to 9,144 gene families with 813 species-specific genes. For these insects, the unique genes ranged from 47 to 3,117, representing their special evolutionary roads, which will be explained in detail using gene family analysis.

Based on the phylogeny reconstruction using single copy genes, all species of Paraneoptera (Psocodea, Hemiptera, Thysanoptera) formed a clade while other insects clustered together. However, several recent studies [26] reported Psocodea might cluster with Holometabola insects, but not with (Hemiptera + Thysanoptera). These could be the results of sampling differences and the gene datasets used for phylogeny inference. Our results, thus, indicated a more tight relationship between Paraneoptera clades. Based on the results of MCMCTREE, the divergence time between booklice and parasitic lice was ~231 MYA, similar to several previous studies [25].

3.4 Gene family expansion and contraction

Based on the results from CAFE, we detected how the gene family evolved in Psocodea, which might account for the formation of parasitism (Fig. 3). For the MRCA of booklouse (*L. brunnea*) and parasitic lice (*P. humanus* and *C. columbae*), 145 gene families expanded while 3,757 contracted. The large number of contracted gene families indicated potential biological

functional loss (Kirkness et al. 2010). Indeed, based on Kinfin results (Table S1), gene families including P450s, G protein-coupled receptors (GPCRs), odorant receptor (OR), gustatory receptor (Gr), CSP contracted in both *P. humanus* and *C. columbae*. Similar gene family (GPCR, P450s) contraction was observed in the MRCA of booklice and parasitic lice, whereas there's a gene family expansion of Gr genes and no change of OR, CSP genes. Such results indicate that the sense-related gene family changed mainly in the parasitic lice, but not booklice. Conversely, the gene families of GPCR, OR, CSP, P450 expanded significantly in *L. brunnea* and this might be explained by the necessity of free-living lifestyle and adaptation to environmental change [71,72].

The BITOCORA analyses (Table 2) confirmed the Kinfin results with the three sensing related gene families (CSP, OR, OBP) contracted in parasitic lice and expanded in *L. brunnea*. Surprisingly, the HSP gene family kept about 40 genes in all three lice while theoretically the host could largely guarantee the body temperature of the parasitic lice. Moreover, we found all resistance related gene families (ABC, EST, GST, UGT and P450) kept their amount in *L. brunnea* while contracted in the two parasitic lice, indicating of a less environmental challenge to the latter clade. This is particular true in several parasitic animal and plant clades [73,74].

3.5 P450 genes in phosphine resistance

Among all resistance related gene families, we noticed the P450 gene family had a very large number with 125 P450 genes (Table 2). Nonetheless, *L. brunnea* has a quite big P450 gene family compared to all other close species. Four P450 subfamilies (CYP2, CYP3, CYP4, Mito) of *L. brunnea* included 13, 44, 50, 16 genes while *F. occidentalis* had 10, 29, 43, 10 genes and *P. humunus* had 7, 12, 11, 10 genes for each subfamily (Fig. 4A). Compared to the

parasitic lice, all four subfamilies of *L. brunnea* expanded significantly. The CYP4 subfamily had the largest number of genes, which could be the potential reason for high insecticides resistance. Similar CYP4 subfamily expansion was observed in *Thrips palmi*, which accounted partially for its high insecticide resistance [75].

We then analysed how P450 genes reacted to phosphine fumigation. After RNA-seq analyses, under the rule of P value <0.05 and mRNA expression fold change >2, we found 11 differential expressed genes (DEGs) from P450 gene family (Fig. 4B, Table S2), distributed in CYP4 (9 genes) and CYP2 (9 genes) subfamilies. The other two DEGs were from the CYP2 subfamily with one upregulated and the other downregulated. As is predicted, most DEGs were from the CYP4 subfamily with seven of them upregulated, suggesting the largest P450 subfamily (CYP4) had the most important biological function for phosphine resistance. Our results indicated that the high fumigant resistance in booklice species might originate from the expansion of the P450 gene family, especially its CYP4 subfamily.

3.6 HSP genes in heat tolerance

Based on the fact that booklice favour high temperatures and the HSP genes functioned during heat treatment across many species, we supposed the free-living booklice should had an expanded HSP gene family. However, our gene family analyses proved the HSP gene family of *L. brunnea* had a small number of genes across all species (Table 2). All three lice had about 40 P450 genes, indicating of a similar evolutionary pathway in Psocodea (Psocoptera+ Phthiraptera). There might be two reasons for the conservation of HSP genes: 1) as the epibiont, parasitic lice still suffered fluctuant temperatures under various host activities; 2) HSP genes are key component of other necessary biological function, i.e. insect sleep [76].

Five HSP subfamilies were identified in *L. brunnea* (Fig. 5A), including HSP20 (5),

HSP40 (8), HSP60 (11), HSP70 (15) and HSP90 (6). After the RNA-seq analysis, we found 8 P450 genes from 4 subfamilies upregulated significantly, indicating the importance of these HSP genes during the heat tolerance of *L. brunnea* (Fig. 5B). HSP genes have been proved to be key in the temperature adaptation in insects [77]. Our findings confirmed these results and provided further evidence on how psocids adapted to this important ecological aspect.

4. Conclusions

Here, we report a high-quality genome assembly of *L. brunnea*, type species in the genus *Liposcelis*. It becomes the first genome from the order Psocoptera uncovered. The genome of *L. brunnea* has a contig N50 of 1.78 Mb and is distributed into nine LGs. The lice clade, including booklice, barklice and parasitic lice diversified for about 231 million years, with sensing and resistance related gene families contracted in the latter clade. We found P450 genes, especially those from CYP4 subfamily, effect during phosphine fumigation, and thus become key genetic targets for genetic based pest control methods. About a quarter HSP genes upregulated under heat treatment, indicating of their importance in temperature adaptation. Overall, our study provided valuable data and insights into lice evolution and environmental adaptation.

Data Accessibility

Illumina DNA/RNA sequencing data, PacBio sequel II genome sequencing and Hi-C data were uploaded at NCBI SRA under BioProject: PRJNA772023. The genome assembly is under NCBI WGS Accession: JAJEOV000000000. The reviewer link of the data is：https://dataview.ncbi.nlm.nih.gov/object/PRJNA772023?reviewer=7lsh5mqhru55qpn1h904r5lav4

Additional Files

Table S1. Annotation of clade-specific expansion/contraction gene families from KinFin analysis.

Table S2. Differential expressed genes in P450 gene family during phosphine fumigation.

Competing Interests

The authors declare that they have no competing interests.

Funding

This work was supported by the Key Research Program of International Collaboration between China and Czech Republic (2018YFE0108700) to Z. L., China Agriculture Research System of MOF and MARA to Z. L.

Authors' Contributions

SF and ZL conceived the project and wrote the manuscript. GO, VS and ZL collected and identified the samples. SF performed the analyses. SF and WD performed the experiments. All authors read and approved the final manuscript.

Acknowledgements

We thank Yueyang Zhou, Dr Qianqian Yang for their help on sample rearing and data analysis.

References

1. Phillips TW, Throne JE. Biorational approaches to managing stored-product insects. *Annu Rev Entomol*. annualreviews.org; 55:375–972010;

2. Nayak MK, Collins PJ, Throne JE, Wang J-J. Biology and management of psocids infesting stored products. *Annu Rev Entomol*. 59:279–972014;

3. Stejskal V, Hubert J, Aulicky R, Kucerova Z. Overview of present and past and pest-associated risks in stored food and feed products: European perspective. *J Stored Prod Res*. Elsevier; 64:122–322015;

4. Athanassiou CG, Rumbos CI. Emerging Pests in Durable Stored Products. In: Athanassiou CG, Arthur FH, editors. *Recent Advances in Stored Product Protection*. Berlin, Heidelberg: Springer Berlin Heidelberg; p. 211–27.

5. Lienhard C, Smithers CN. Psocoptera (Insecta): World catalogue and bibliography. cabdirect.org; 2002;

6. Grimaldi D, Engel MS. Fossil Liposcelididae and the lice ages (Insecta: Psocodea). *Proc Biol Sci*. royalsocietypublishing.org; 273:625–332006;

7. Turner BD. Forming a clearer view of L. bostrychophilus. *Environ Health*. 95:9–131987;

8. Macfarlane JA, Ja M. Damage to milled rice by psocids. pascal-francis.inist.fr; 1982;

9. Kučerová Z. Weight losses of wheat grains caused by psocid infestation. *Plant Prot Sci*. 81.0.228.28; 38:103–72002;

10. Turner BD, Staines NA, Brostoff J, Howe CA, Cooper K, Wildey KB. Allergy to psocids. *Proceedings of the International Conference on Insect Pests in the Urban Environment (ICIPUE)*. ICIPUE, Heriot-Watt University Edinburgh, Scotland; p. 7–10.

11. Hubert J, Stejskal V, Athanassiou CG, Throne JE. Health Hazards Associated with Arthropod Infestation of Stored Products. *Annu Rev Entomol*. annualreviews.org; 63:553–732018;

12. Turner BD. Liposcelis bostrychophila (Psocoptera: Liposcelididae), a stored food pest in the UK. *Int J Pest Manage*. Taylor & Francis; 40:179–901994;

13. Nayak MK, Collins PJ, Reid SR. Efficacy of Grain Protectants and Phosphine Against Liposcelis bostrychophila, L. entomophila, and L. paeta (Psocoptera: Liposcelidae). *J Econ*. academic.oup.com; 1998;

14. Daglish GJ, Wallbank BE, Nayak MK. Synergized bifenthrin plus chlorpyrifos-methyl for control of beetles and psocids in sorghum in Australia. *J Econ Entomol*. academic.oup.com; 96:525–322003;

15. Nayak MK, Daglish GJ. Potential of imidacloprid to control four species of psocids (Psocoptera: Liposcelididae) infesting stored grain. *Pest Manag Sci*. Wiley Online Library; 62:646–502006;

16. Athanassiou CG, Arthur FH, Opit GP, Throne JE. Insecticidal effect of diatomaceous earth against three species of stored-product psocids on maize, rice, and wheat. *J Econ Entomol*. academic.oup.com; 102:1673–802009;

17. Pike V. Laboratory assessment of the efficacy of phosphine and methyl bromide fumigation against all life stages of Liposcelis entomophilus (Enderlein). *Crop Prot*. Elsevier; 13:141–51994;

18. Cao Y, Song Y, Sun GY. A survey of psocid species infesting stored grain in China and resistance to phosphine in field populations of Liposcelis entomophila (Enderlein)(Psocoptera …. *product protection Proceedings of the 8th …*. cabdirect.org; 2003;

19. Nayak MK, Collins PJ, Pavic H, Kopittke RA. Inhibition of egg development by phosphine in the cosmopolitan pest of stored products Liposcelis bostrychophila (Psocoptera: Liposcelididae). *Pest Manag Sci*. Wiley Online Library; 59:1191–62003;

20. Wei D-D, He W, Miao Z-Q, Tu Y-Q, Wang L, Dou W, et al.. Characterization of Esterase Genes Involving Malathion Detoxification and Establishment of an RNA Interference Method in Liposcelis bostrychophila. *Front Physiol*. 11:2742020;

21. Wu S, Dou W, Wu J-J, Wang J-J. Purification and partial characterization of glutathione S-transferase from insecticide-resistant field populations of Liposcelis paeta Pearman (Psocoptera: Liposcelididae). *Arch Insect Biochem Physiol*. 70:136–502009;

22. Wei DD, Li T, Chen SC, Dou W, Wang JJ. Molecular studies of psocids in China: recent advances. *proceedings of the 11th International Working Conference on Stored Product Protection 79*. spiru.cgahr.ksu.edu;

23. Boivin V, Reulet G, Boisvert O, Couture S, Elela SA, Scott MS. Reducing the structure bias of RNA-Seq reveals a large number of non-annotated non-coding RNA. *Nucleic Acids Res*. 48:2271–862020;

24. Lyal CHC. Phylogeny and classification of the Psocodea, with particular reference to the lice (Psocodea: Phthiraptera). *Syst Entomol*. John Wiley & Sons, Ltd; 10:145–651985;

25. Johnson KP, Dietrich CH, Friedrich F, Beutel RG, Wipfler B, Peters RS, et al.. Phylogenomics and the evolution of hemipteroid insects. *Proc Natl Acad Sci U S A*. 115:12775–802018;

26. de Moya RS, Yoshizawa K, Walden KKO, Sweet AD, Dietrich CH, Johnson KP. Phylogenomics of parasitic and non-parasitic lice (Insecta: Psocodea): Combining sequence data and Exploring compositional bias solutions in Next Generation Datasets. *Syst Biol*. 2020; doi: 10.1093/sysbio/syaa075.

27. Broadhead E. Revision of the genus Liposcelis Motschulsky with notes on the position of this genus in the order Corrodentia and on the variability of ten liposcelis species. *Transactions of the Royal Entomological Society of*. cabdirect.org; 1950;

28. Mockford EL. Psocoptera from sleeping nests of the dusky-footed wood rat in southern California (Psocoptera: Atropidae, Psoquillidae, Liposcelidae). *Pan pacific Entomol*. agris.fao.org; 1971;

29. Baz A, Others. Psocoptera from weaver bird nests (Aves: Ploceidae) in Equatorial Guinea (West-Africa). *Annales de la Société entomologique de France*. cabdirect.org; p. 33–8.

30. Kirkness EF, Haas BJ, Sun W, Braig HR, Perotti MA, Clark JM, et al.. Genome sequences of the human body louse and its primary endosymbiont provide insights into the permanent parasitic lifestyle. *Proc Natl Acad Sci U S A*. National Acad Sciences; 107:12168–732010;

31. Baldwin-Brown JG, Villa SM, Vickrey AI, Johnson KP, Bush SE, Clayton DH, et al.. The assembled and annotated genome of the pigeon louse Columbicola columbae, a model ectoparasite. *G3* . academic.oup.com; 2021; doi: 10.1093/g3journal/jkab009.

32. Marçais G, Kingsford C. A fast, lock-free approach for efficient parallel counting of occurrences of k-mers. *Bioinformatics*. academic.oup.com; 27:764–702011;

33. Ranallo-Benavidez TR, Jaron KS, Schatz MC. GenomeScope 2.0 and Smudgeplot for reference-free profiling of polyploid genomes. *Nat Commun*. nature.com; 11:14322020;

34. Koren S, Walenz BP, Berlin K, Miller JR, Bergman NH, Phillippy AM. Canu: scalable and accurate long-read assembly via adaptive k-mer weighting and repeat separation. *Genome Res*. genome.cshlp.org; 27:722–362017;

35. Li H. Aligning sequence reads, clone sequences and assembly contigs with BWA-MEM. *arXiv preprint arXiv:13033997*. arxiv.org; 2013;

36. Walker BJ, Abeel T, Shea T, Priest M, Abouelliel A, Sakthikumar S, et al.. Pilon: an integrated tool for comprehensive microbial variant detection and genome assembly improvement. *PLoS One*. journals.plos.org; 9:e1129632014;

37. Guan D, McCarthy SA, Wood J, Howe K, Wang Y, Durbin R. Identifying and removing haplotypic duplication in primary genome assemblies. *Bioinformatics*. academic.oup.com; 36:2896–82020;

38. Durand NC, Shamim MS, Machol I, Rao SSP, Huntley MH, Lander ES, et al.. Juicer Provides a One-Click System for Analyzing Loop-Resolution Hi-C Experiments. *Cell Syst*. Elsevier; 3:95–82016;

39. Dudchenko O, Batra SS, Omer AD, Nyquist SK, Hoeger M, Durand NC, et al.. De novo assembly of the Aedes aegypti genome using Hi-C yields chromosome-length scaffolds. *Science*. science.sciencemag.org; 356:92–52017;

40. Durand NC, Robinson JT, Shamim MS, Machol I, Mesirov JP, Lander ES, et al.. Juicebox Provides a Visualization System for Hi-C Contact Maps with Unlimited Zoom. *Cell Syst*. Elsevier; 3:99–1012016;

41. Seppey M, Manni M, Zdobnov EM. BUSCO: Assessing Genome Assembly and Annotation Completeness. *Methods Mol Biol*. Springer; 1962:227–452019;

42. Flynn JM, Hubley R, Goubert C, Rosen J, Clark AG, Feschotte C, et al.. RepeatModeler2 for automated genomic discovery of transposable element families. *Proc Natl Acad Sci U S A*. National Acad Sciences; 117:9451–72020;

43. Chen N. Using RepeatMasker to identify repetitive elements in genomic sequences. *Curr Protoc Bioinformatics*. Wiley; Chapter 4:Unit 4.102004;

44. Campbell MS, Holt C, Moore B. Genome annotation and curation using MAKER and MAKER‐ P. *Current protocols in*. Wiley Online Library; 2014;

45. Grabherr MG, Haas BJ, Yassour M, Levin JZ, Thompson DA, Amit I, et al.. Full-length transcriptome assembly from RNA-Seq data without a reference genome. *Nat Biotechnol*. nature.com; 29:644–522011;

46. Camacho C, Coulouris G, Avagyan V, Ma N, Papadopoulos J, Bealer K, et al.. BLAST+: architecture and applications. *BMC Bioinformatics*. Springer; 10:4212009;

47. Slater GSC, Birney E. Automated generation of heuristics for biological sequence comparison. *BMC Bioinformatics*. Springer; 6:312005;

48. Korf I. Gene finding in novel genomes. *BMC Bioinformatics*. bmcbioinformatics.biomedcentral …; 5:592004;

49. Stanke M, Morgenstern B. AUGUSTUS: a web server for gene prediction in eukaryotes that allows user-defined constraints. *Nucleic Acids Res*. academic.oup.com; 33:W465-72005;

50. Buchfink B, Reuter K, Drost H-G. Sensitive protein alignments at tree-of-life scale using DIAMOND. *Nat Methods*. nature.com; 18:366–82021;

51. Zdobnov EM, Apweiler R. InterProScan–an integration platform for the signature-recognition methods in InterPro. *Bioinformatics*. academic.oup.com; 2001;

52. Huerta-Cepas J, Forslund K, Coelho LP, Szklarczyk D, Jensen LJ, von Mering C, et al.. Fast Genome-Wide Functional Annotation through Orthology Assignment by eggNOG-Mapper. *Mol Biol Evol*. academic.oup.com; 34:2115–222017;

53. Emms DM, Kelly S. OrthoFinder: phylogenetic orthology inference for comparative genomics. *Genome Biol*. genomebiology.biomedcentral.com; 20:2382019;

54. Katoh K, Standley DM. MAFFT multiple sequence alignment software version 7: improvements in performance and usability. *Mol Biol Evol*. academic.oup.com; 30:772–802013;

55. Yang Z. PAML 4: phylogenetic analysis by maximum likelihood. *Mol Biol Evol*. academic.oup.com; 24:1586–912007;

56. De Bie T, Cristianini N, Demuth JP, Hahn MW. CAFE: a computational tool for the study of gene family evolution. *Bioinformatics*. academic.oup.com; 22:1269–712006;

57. Laetsch DR, Blaxter ML. KinFin: Software for Taxon-Aware Analysis of Clustered Protein Sequences. *G3* . academic.oup.com; 7:3349–572017;

58. Finn RD, Clements J, Eddy SR. HMMER web server: interactive sequence similarity searching. *Nucleic Acids Res*. academic.oup.com; 39:W29-372011;

59. Wickham H. Ggplot2. *Wiley Interdiscip Rev Comput Stat*. Wiley; 3:180–52011;

60. Team RC, Others. R: A language and environment for statistical computing. Vienna, Austria; 2013;

61. Vizueta J, Sánchez-Gracia A, Rozas J. bitacora: A comprehensive tool for the identification and annotation of gene families in genome assemblies. *Mol Ecol Resour*. Wiley Online Library; 20:1445–522020;

62. Edgar RC. MUSCLE: multiple sequence alignment with high accuracy and high throughput. *Nucleic Acids Res*. academic.oup.com; 32:1792–72004;

63. Kim D, Paggi JM, Park C, Bennett C, Salzberg SL. Graph-based genome alignment and genotyping with HISAT2 and HISAT-genotype. *Nat Biotechnol*. nature.com; 37:907–152019;

64. Liao Y, Smyth GK, Shi W. featureCounts: an efficient general purpose program for assigning sequence reads to genomic features. *Bioinformatics*. Oxford Academic; 30:923–302014;

65. Robinson MD, McCarthy DJ, Smyth GK. edgeR: a Bioconductor package for differential expression analysis of digital gene expression data. *Bioinformatics*. academic.oup.com; 26:139–402010;

66. Li F, Zhao X, Li M, He K, Huang C, Zhou Y, et al.. Insect genomes: progress and challenges. *Insect Mol Biol*. 28:739–582019;

67. Kidwell MG. Transposable elements and the evolution of genome size in eukaryotes. *Genetica*. Springer; 115:49–632002;

68. Haubold B, Wiehe T. How repetitive are genomes? *BMC Bioinformatics*. 7:5412006;

69. Dennis AB, Ballesteros GI, Robin S, Schrader L, Bast J, Berghöfer J, et al.. Functional insights from the GC-poor genomes of two aphid parasitoids, Aphidius ervi and Lysiphlebus fabarum. *BMC Genomics*. BioMed Central; 21:1–272020;

70. Lefébure T, Morvan C, Malard F, François C, Konecny-Dupré L, Guéguen L, et al.. Less effective

selection leads to larger genomes. *Genome Res*. 27:1016–282017;

71. Brand P, Ramírez SR. The Evolutionary Dynamics of the Odorant Receptor Gene Family in Corbiculate Bees. *Genome Biol Evol*. 9:2023–362017;

72. Ritschard EA, Fitak RR, Simakov O, Johnsen S. Genomic signatures of G-protein-coupled receptor expansions reveal functional transitions in the evolution of cephalopod signal transduction. *Proc Biol Sci*. 286:201829292019;

73. Sun G, Xu Y, Liu H, Sun T, Zhang J, Hettenhausen C, et al.. Large-scale gene losses underlie the genome evolution of parasitic plant Cuscuta australis. *Nat Commun*. 9:26832018;

74. ChuanLin Y, XinHai Y, MengYao C, Yang M, HuaMei X, Fei L. Evolution analysis of cytochrome P450 gene family in parasitoid wasps. *Zhongguo Sheng Wu Fang Zhi*. Editorial Board of Chinese Journal of Biological Control; 35:335–422019;

75. Guo S, Cao L, Song W, Shi P, Gao Y, Gong Y, et al.. Chromosome- level assembly of the melon thrips genome yields insights into evolution of a sap- sucking lifestyle and pesticide resistance. *Mol Ecol Resour*. Wiley Online Library; 20:1110–252020;

76. Zhao X, Li Y, Zhao Z, Du J. Extra sex combs buffers sleep- related stresses through regulating Heat shock proteins. *The FASEB Journal*. Wiley Online Library; 2021;

77. González-Tokman D, Córdoba-Aguilar A, Dáttilo W, Lira-Noriega A, Sánchez-Guillén RA, Villalobos F. Insect responses to heat: physiological mechanisms, evolution and ecological implications in a warming world. *Biol Rev Camb Philos Soc*. Wiley; 95:802–212020;

Tables

Table 1. Published genomes in Psocodea.

Genome Features	Parasitic Lice		Booklice
	Pediculus humanus	*Columbicola columbae*	*Liposcelis brunnea*
Genome Size (MB)	110	208	174
Chromosomes	6	12	9
Methods	Capillary Platform	Nanopore+Illumina+HiC	Pacbio+Illumina+HiC
Contig N50	-	511 kb	1.78 Mb
Scaffold N50	488 kb	17.6 Mb	19.7 Mb
Genes	10,773	13,362	15,543
Repetitive elements	7.3% (8.0 Mb)	9.7% (20.2 Mb)	15.9% (27.7 Mb)
BUSCO Evaluation	95.9%	96.4%	97.2%

Table 2. Gene families in representative insect species.

Gene Family	Psocodea			Hemiptera			Thysanoptera		Diptera
	Pediculus humunus	*Columbicola columbae*	*Liposcelis brunnea*	*Acyrthosiphon pisum*	*Bemisia tabaci*	*Nilaparvata lugens*	*Frankliniella occidentalis*	*Thrips palmi*	*Drosophila melanogaster*
ABC	43	47	66	107	53	80	65	57	57
EST	26	29	69	45	49	81	66	78	42
GST	24	18	44	34	32	26	33	36	54
UGT	4	3	19	60	80	20	26	18	35
P450	43	44	125	79	141	88	95	115	92
HSP	41	39	42	48	44	71	71	63	58
CSP	6	8	9	10	19	17	11	11	4
OBP	3	5	37	18	8	23	16	24	47
OR	10	9	29	17	10	31	13	15	67

Figures

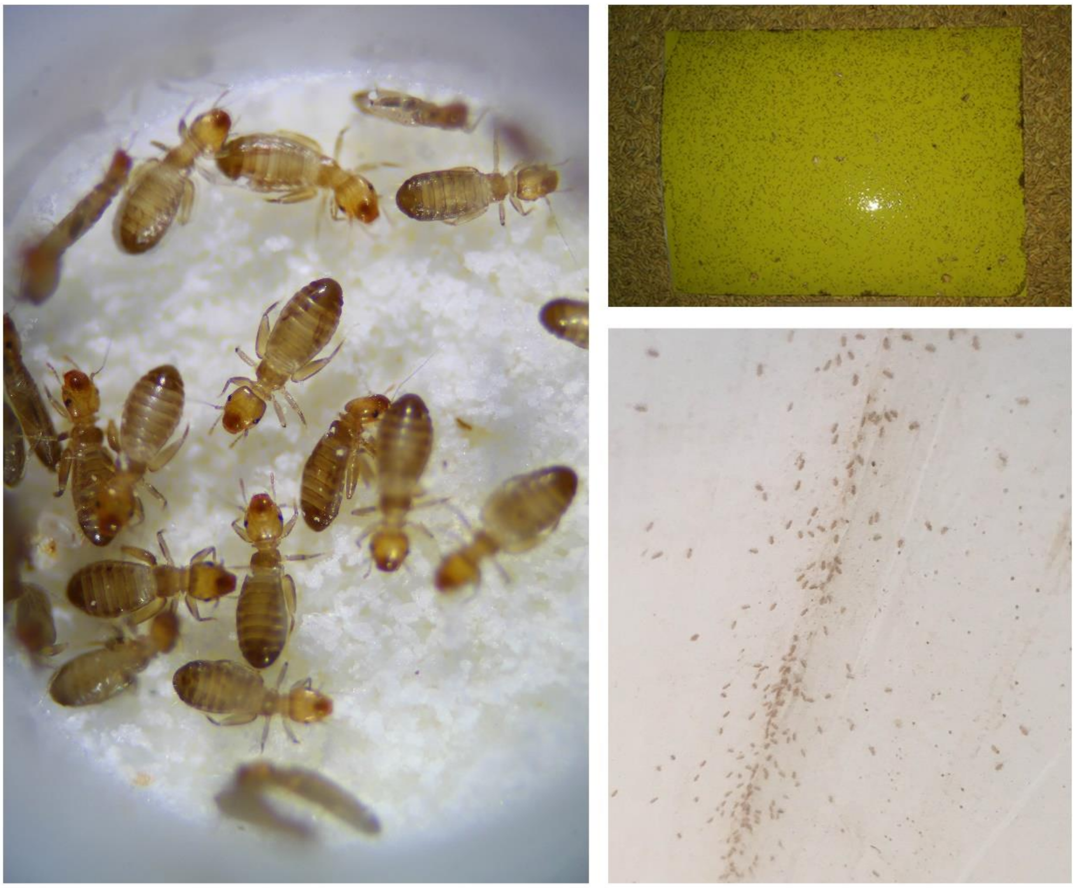

Figure 1. The adult booklouse, *Liposcelis brunnea* in the present study. They are reared in artificial diet in the lab (left figure). They are always trapped in the grain storage, especially the corner of storage facilities (right figures).

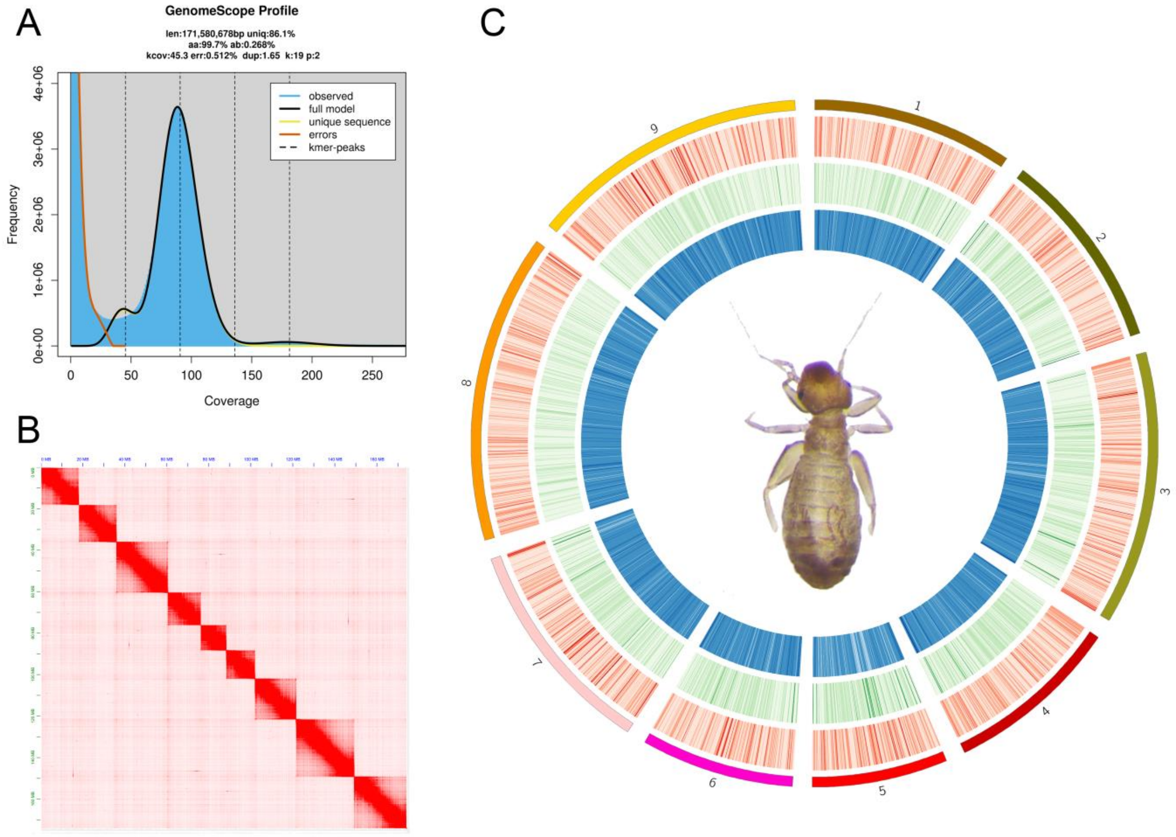

Figure 2. Genome survey, HiC and basic genomic features. (a) GenomeScope estimation of genome size, heterogeneity using a k-mer of 19. (b) HiC interaction map produced by 3D-DNA. (c) Circos plot of the linkage groups. The outer 9 bars represented 9 linkage groups while the red, green, blue heat maps represented gene counts, repeat sequences and GC content in 50 kb windows.

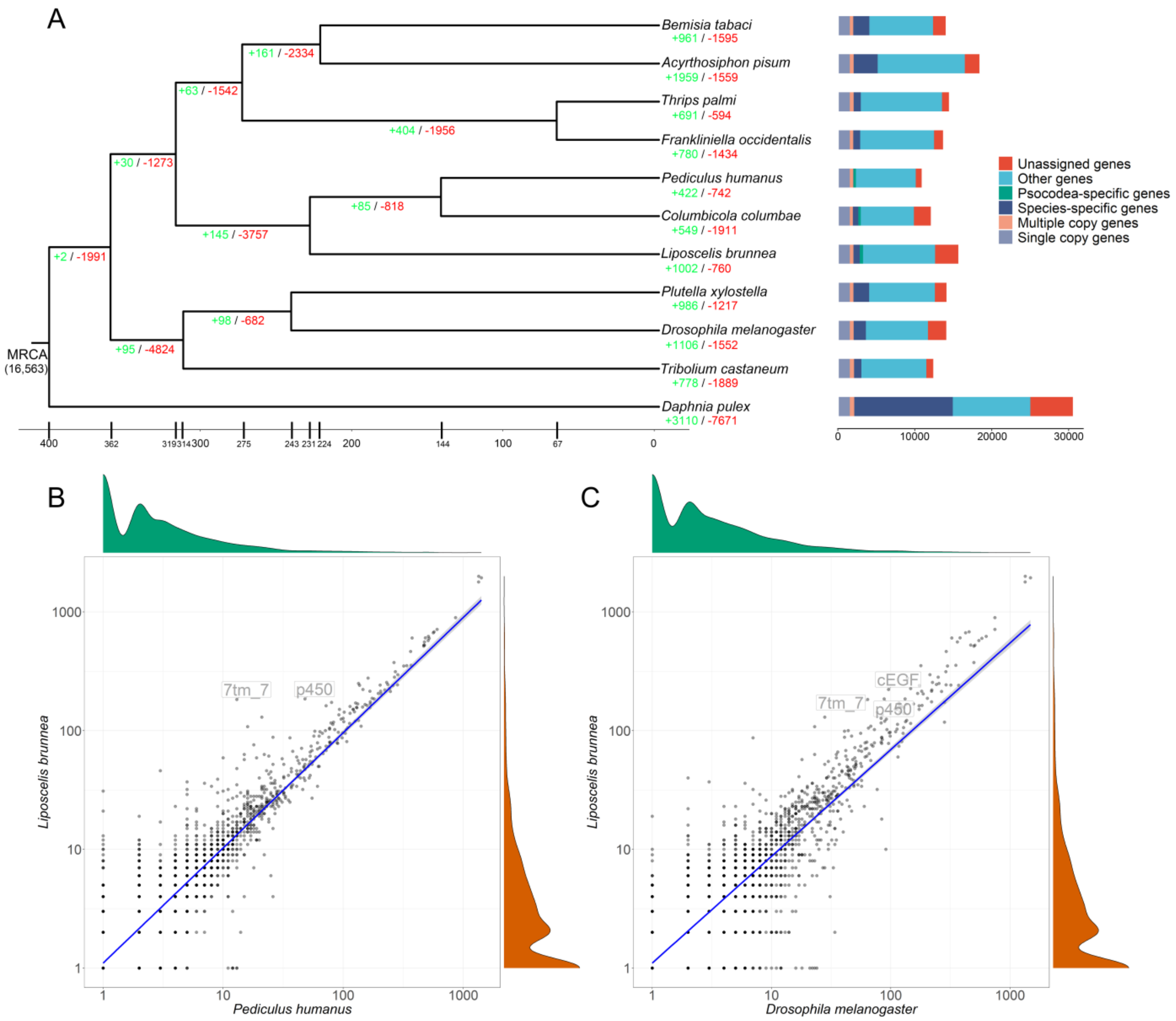

Figure 3. Gene family evolution among *L. brunnea* and other insects. (a) Phylogenetic trees inferred from concatenated single copy genes using Fasttree. MCMCTREE was used for molecular dating. The single copy genes, multiple copy genes, species specific genes, clade specific genes were analyzed based on the results from OrthoFinder. Gene family expansion (green) and contraction (red) was analyzed using CAFE. (b) HMMER based protein domain comparisons. The non-redundant proteins of *L. brunnea* were compared against *P. humanus* and *D. melanogaster*. Several significantly expanded gene families were labelled, for example, P450 (p450), odorant and gustatory receptor (7tm_7) families.

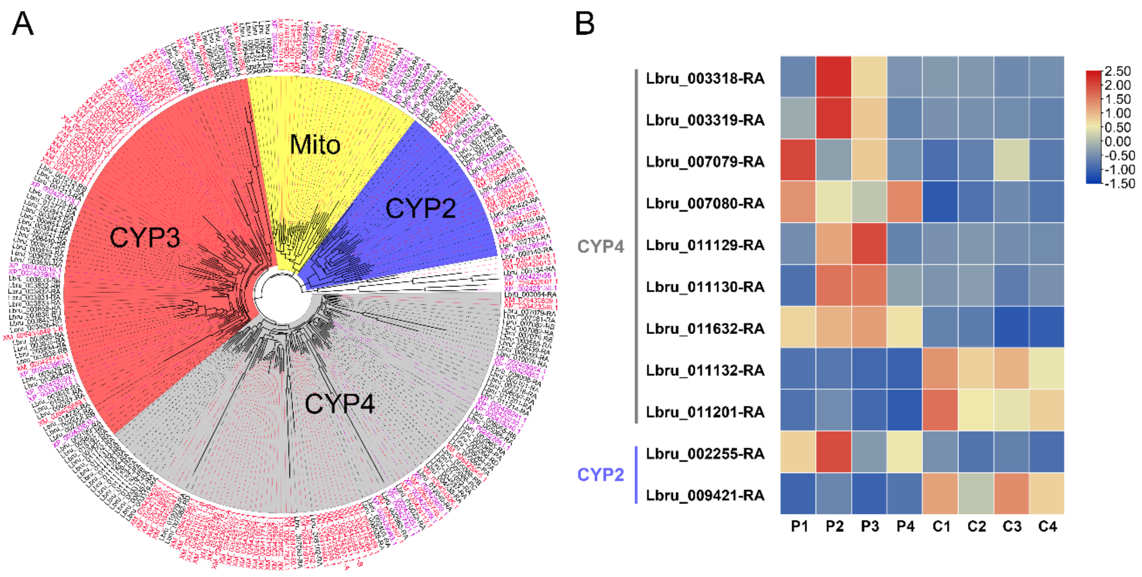

Figure 4. Phylogenetic tree of P450 gene family and significantly expressed genes. (A) The P450 genes of *L. brunnea* (in black), *P. humanus* (in purple), *D. melanogaster* (in red) from BITACORA analysis were used to construct a neighbor-joining tree where four subfamilies separated obviously. (B) Totally four treatment (P1-P4) and four control (C1-C4) groups were analysed. The subfamilies of 11 differential expressed P450 genes were listed. The expression data was normalized for each gene.

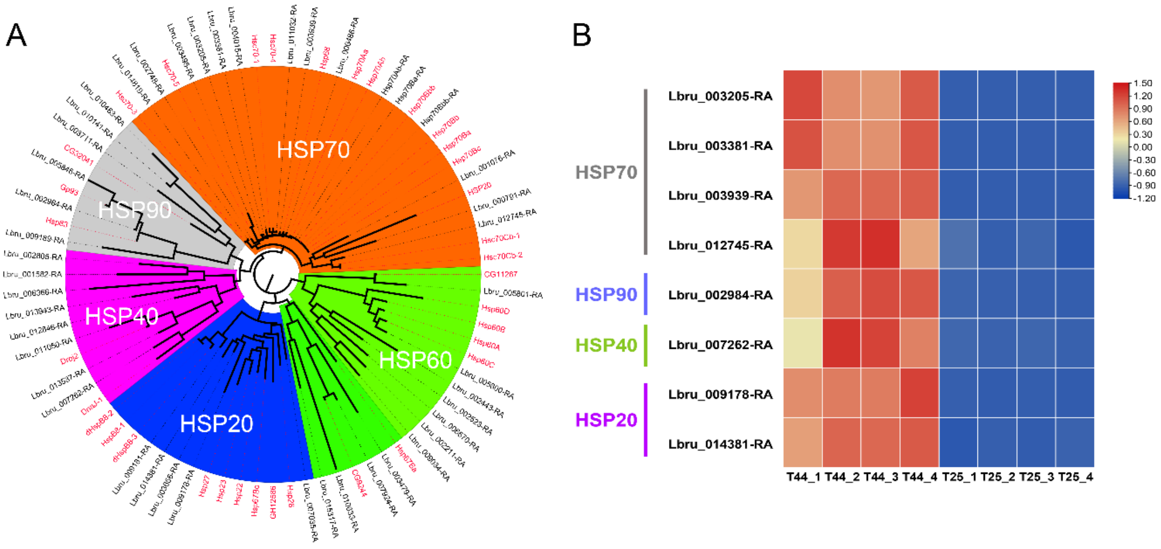

Figure 5. Phylogenetic tree of HSP gene family and significantly expressed genes. (A) The HSP genes of *L. brunnea* (in black) and *D. melanogaster* (in red) from BITACORA analysis were used to construct a neighbor-joining tree where five subfamilies separated obviously. (B) Totally four treatment (T44_1-T44_4) and four control (T25_1-T25_4) groups were analysed. The subfamilies of 8 differential expressed HSP genes were listed. The expression data was normalized for each gene.

Table S1

Click here to access/download
Supplementary Material
Table S1.xlsx

Table S2

Click here to access/download
Supplementary Material
Table S2.xlsx
