# Supplementary material for: A chromosome-level genome of the booklouse, Liposcelis brunnea, provides insight into louse evolution and environmental stress adaptation
Source: Gigascience. 2022 Jul 19;11:giac062. doi: 10.1093/gigascience/giac062 (PMC9295366; doi:10.1093/gigascience/giac062)
Supplement: giac062_GIGA-D-22-00060_Revision_1 [file giac062_giga-d-22-00060_revision_1.pdf]

GigaScience

A chromosome-level genome of the booklouse, Liposcelis brunnea provides insight into louse evolution and environmental stress adaptation

--Manuscript Draft--

**Manuscript Number:**	GIGA-D-22-00060R1
**Full Title:**	A chromosome-level genome of the booklouse, Liposcelis brunnea provides insight into louse evolution and environmental stress adaptation
**Article Type:**	Data Note
**Funding Information:**	Key Research Program of International Collaboration between China and Czech Republic (2018YFE0108700) — Professor Zhihong Li<br>China Agriculture Research System of MOF and MARA — Professor Zhihong Li
**Abstract:**	Background: Booklice (psocids) in the genus Liposcelis (Psocoptera: Liposcelididae), are a group of important storage pests, found in libraries, grain storages and food processing facilities. Booklice are able to survive under heat treatment and typically possess high resistance to common fumigant insecticides, hence posing a threat to storage security worldwide.Results: We assembled the genome of the booklouse, L. brunnea, the first genome reported in Psocoptera, using PacBio long-read sequencing, Illumina sequencing and chromatin conformation capture (HiC) methods. After assembly, polishing, haplotype purging and HiC scaffolding, we obtained nine linkage groups (174.1 Mb in total) ranging from 12.1 Mb to 27.6 Mb (N50: 19.7 Mb), with the BUSCO completeness at 98.9%. In total 15,543 genes were predicted by the Maker pipeline. Gene family analyses indicated the sensing-related gene families (OBP and OR) and the resistance-related gene families (ABC, EST, GST, UGT and P450) expanded significantly in L. brunnea compared with those of their closest relatives (two parasitic lice). Based on transcriptomic analysis, we found that the CYP4 subfamily from the P450 gene family functioned during phosphine fumigation; HSP genes, particularly those from the HSP70 subfamily, were upregulated significantly under high temperatures.Conclusions: We present a chromosome-level genome assembly of L. brunnea, the first genome reported for the order Psocoptera. Our analyses provide new insights into the gene family evolution of the louse clade and the transcriptomic responses of booklice to environmental stresses.
**Corresponding Author:**	Shiqian Feng<br>China Agricultural University<br>Beijing, CHINA
**Corresponding Author Secondary Information:**	
**Corresponding Author's Institution:**	China Agricultural University
**Corresponding Author's Secondary Institution:**	
**First Author:**	Shiqian Feng
**First Author Secondary Information:**	
**Order of Authors:**	Shiqian Feng<br>George Opit<br>Wenxin Deng<br>Vaclav Stejskal<br>Zhihong Li
**Order of Authors Secondary Information:**	
**Response to Reviewers:**	We thank the editor’s help for processing our manuscript. We thank two reviewers for

Powered by Editorial Manager® and ProduXion Manager® from Aries Systems Corporation

their valuable comments on our work. We believe that through their comments, we were able to significantly improve our work. We have responded to each of the points made by the reviewers here. We hope you will find the revised version of the manuscript improved, and suitable for publication. We submitted the clean modified manuscript file and the file with changes tracked. We specified all the modifications in the response letter and please don't hesitate to contact us if there are any ambiguous points or files necessary for our manuscript evaluation.

Reviewer #1:
This manuscript by Feng et al. titled "A chromosome-level genome of the booklouse, Liposcelis brunnea provides insight into lice evolution and environmental stress adaptation" provides useful information on the genome organization, transcriptome, and some aspects of physiology. Authors have used technology typically used for sequencing, assembling, scaffolding, and annotating genomes and satisfactorily achieved their goals. The manuscript is sufficiently descriptive and easy to follow with sufficient technical details and references to appropriate manuscripts. They have provided parameters used in various analysis steps when deviated from defaults. Overall, the manuscript is in very good shape except for several sentence structure and English language issues which can be corrected easily with professional assistance.

Response: We thank the reviewer for their interest in our paper and the valuable insights. We have conducted language polish by all the authors and with professional assistance from American Journal Experts company.

Please see some of the issues I believe that needs to be fixed:
Line 24: The sentence starting with "Totally 15,543 genes..." may be revised to include written language.
Line 31: Conclusions: Insufficient details. Filling in the blanks? I think you can do a better job than this.
Line 49: Word "which" is duplicated.
Line 175: "For each gene family, we manually annotated five gene resistance related families..." Not sure what authors meant by "five gene resistance related families." First, the authors should define what type of resistance they are discussing here. Is it resistance to corrosion? I guess not. I am sure the authors are referring to insecticide resistance. Please revise the sentences to make sense and be specific.

Response: We modified "Totally" to "In total" (Line 25 in Main_Manuscript_changes_tracked.docx) and supplemented necessary content in the Abstract/Conclusions (Lines 33-37). The duplicated "which" was removed (Line 55) and the descriptive word "insecticide" was added before "resistance" (Line 204).

Table 1: Title needs to be more descriptive to indicate what is shown in this table.
Table 2: Title is too short and not descriptive enough to indicate what is shown in this table.

Response: We changed the titles of two tables (Lines 772, 774) and made them more descriptive.

Figure 2: Figures are too small. Hope the digital version is of high resolution and readable.
Figure 2 title: "...repeat sequences and GC content in 50 kb windows." should be "...repeat sequences and GC content, respectively, in 50 kb windows."
Figures 4A and 5A: Too small and crammed to be a useful figure. Needs to make it substantially large.
Above are only a representative sample of revisions required to the manuscript text to improve the readability.

Response: We attached the high-resolution figures of Figure 2, 4, 5 and we believe these figures would solve reviewer #1's figure-related questions. The title of Figure 2 was modified as suggested (Line 788). We thank reviewer #1 for the valuable and detailed advices.

Reviewer #2:
Booklice is a group of important storage pests that can live under heat treatment and usually possess high resistance to common fumigant insecticides. This paper reported the first genome in Psocoptera with high quality, using PacBio long-read sequencing, Illumina sequencing and HiC methods, and was well annotated. Gene family analyses and transcriptomic analysis provide new insights into lice evolution and the strategy of how insects adapt to environmental stresses. This study combines experimental biology evidence and provides valuable data to provide new insights for future studies. Here are my comments:

Response: We thank the reviewer for their interest and comment of our work.

Line 24, Totally, change to, in total
Line 190, ppm, change to, mg/L
Line 190, methods of treating the adults need to be described in detail. How long time did you treat the adults before the collection of samples?

Response: We modified the manuscript following the reviewer's suggestions (Lines 25, 220 in Main_Manuscript_changes_tracked.docx). The words were modified accordingly and the fumigation/high temperature treatments were detailly described (Lines 220-223).

In Samples collection, feeding conditions and feed ingredients are introduced. I suggest adding sampling time, breeding generation and identification method, which may help to explain the high heterozygosity of the draft genome. Furthermore, I hope the authors describe why only the females were selected for sequencing and transcriptome analysis.

Response: We added the information of sampling time, breeding generation and identification traits of Liposcelis brunnea (Lines 94-97). For the heterozygosity, the evaluated value is 0.268%, which should be relatively small across Insecta. We believe the redundant contigs during the assembly process is mainly from the parameters of Canu, where "correctedErrorRate=0.035" is a quite critical value to separate similar sequences, i.e., Canu is prone to keep the contigs even when they have only a few SNPs. This is also a try to see if we can assemble the diploid genome directly. However, nothing we could do with only these redundant contigs, so we chose to purge them as most researchers and studies have done. We also supplemented the reason why we use females (Lines 103-105), that is, L. brunnea might have a sex determination pattern of XO, meaning we can assemble the whole set of chromosomes using either females or males. Females have a large body size and more DNA, so we can use minimum insects for sequencing which also help eliminate the meaningless heterozygosity.

In the part of Methods, there is much software in this part which needs to be checked carefully. Some software lacks versions or literature information, such as "Juicer" in line 125 and "Juicebox" in 126 lack versions information, and in line 157 "Fasttree" lacks reference information.

Response: We followed the reviewer's suggestions and supplemented the version, references of the software. Moreover, we carefully checked all software in Methods part and added version and reference information.

In the part of Transcriptome analysis under phosphine fumigation/high temperature, there is no definite basis for phosphine concentration and temperature. This process may affect the number and type of differential expressed genes. I hope the author can explain why they choose this concentration and temperature.

Response: In this study, we would like to check the initial transcriptomic response of L. brunnea to the stresses, so we set the treatment time at 2 hours. There is no direct phosphine test of L. brunnea, thus we used the data from a close species, L. bostrychophila as the reference. Mangoba et al. (2021) found a complete control of L. bostrychophila at 0.1-0.5 mg/L for 72h. So we chose to take a less lethal concentration at 0.075 mg/L. For temperature related study, we want to have an extraordinarily high temperature treatment. Pre-experiments showed all L. brunnea individuals survived

Powered by Editorial Manager® and ProduXion Manager® from Aries Systems Corporation

	after 2h treatment under 44℃, which also satisfied our requirement of alive samples before RNA extraction.<br><br>Reference: Mangoba, M. A. A., & de Guzman Alvindia, D. (2021). Phosphine Resistance in Psocid, Liposcelis bostrychophila (Psocoptera) in the Philippines. International Journal of Tropical Insect Science, 41(1), 439–445.<br><br>Furthermore, we went through the manuscript once again to check and remove typos and correct spelling errors.<br><br>Response: We go over the manuscript once again and check the grammar and spellings. We also used a language modification service from the American Journal Experts to better standardize our manuscript. Thanks for the reviewer's suggestions, which helped improve our manuscript greatly.
**Additional Information:**	
**Question**	**Response**
Are you submitting this manuscript to a special series or article collection?	No
**Experimental design and statistics**<br><br>Full details of the experimental design and statistical methods used should be given in the Methods section, as detailed in our Minimum Standards Reporting Checklist. Information essential to interpreting the data presented should be made available in the figure legends.<br><br>Have you included all the information requested in your manuscript?	Yes
**Resources**<br><br>A description of all resources used, including antibodies, cell lines, animals and software tools, with enough information to allow them to be uniquely identified, should be included in the Methods section. Authors are strongly encouraged to cite Research Resource Identifiers (RRIDs) for antibodies, model organisms and tools, where possible.<br><br>Have you included the information requested as detailed in our Minimum Standards Reporting Checklist?	Yes
**Availability of data and materials**	Yes

All datasets and code on which the conclusions of the paper rely must be either included in your submission or deposited in publicly available repositories (where available and ethically appropriate), referencing such data using a unique identifier in the references and in the "Availability of Data and Materials" section of your manuscript.<br><br>Have you have met the above requirement as detailed in our Minimum Standards Reporting Checklist?	

Manuscript

Click here to access/download;Manuscript;Main_Manuscript_final.docx

A chromosome-level genome of the booklouse, *Liposcelis brunnea* provides insight into louse evolution and environmental stress adaptation

Shiqian Feng[1,2], George Opit[3], Wenxin Deng[1,2], Vaclav Stejskal[4,5], Zhihong Li[1,2,*]

1. Department of Plant Biosecurity, College of Plant Protection, China Agricultural University, Beijing 100193, China

2. Key Laboratory of Surveillance and Management for Plant Quarantine Pests, Ministry of Agriculture and Rural Affairs, Beijing 100193, China

3. Department of Entomology and Plant Pathology, Oklahoma State University, Oklahoma 74078, USA

4. Crop Research Institute, Drnovská 507, 161 06 Prague 6, Czech Republic

5. Czech University of Life Sciences, Faculty of Agrobiology, Food and Natural Resources, Kamycka 129, 165 00 Prague, Czech Republic

* Corresponding author: lizh@cau.edu.cn

Shiqian Feng [0000-0002-8495-6629];

Vaclav Stejskal [0000-0002-0192-3275];

Zhihong Li [0000-0002-1281-2108]

Abstract

Background: Booklice (psocids) in the genus *Liposcelis* (Psocoptera: Liposcelididae), are a group of important storage pests, found in libraries, grain storages and food processing facilities. Booklice are able to survive under heat treatment and typically possess high resistance to common fumigant insecticides, hence posing a threat to storage security worldwide.

Results: We assembled the genome of the booklouse, *L. brunnea*, the first genome reported in Psocoptera, using PacBio long-read sequencing, Illumina sequencing and chromatin conformation capture (HiC) methods. After assembly, polishing, haplotype purging and HiC scaffolding, we obtained nine linkage groups (174.1 Mb in total) ranging from 12.1 Mb to 27.6 Mb (N50: 19.7 Mb), with the BUSCO completeness at 98.9%. In total 15,543 genes were predicted by the Maker pipeline. Gene family analyses indicated the sensing-related gene families (OBP and OR) and the resistance-related gene families (ABC, EST, GST, UGT and P450) expanded significantly in *L. brunnea* compared with those of their closest relatives (two parasitic lice). Based on transcriptomic analysis, we found that the CYP4 subfamily from the P450 gene family functioned during phosphine fumigation; HSP genes, particularly those from the HSP70 subfamily, were upregulated significantly under high temperatures.

Conclusions: We present a chromosome-level genome assembly of *L. brunnea*, the first genome reported for the order Psocoptera. Our analyses provide new insights into the gene family evolution of the louse clade and the transcriptomic responses of booklice to environmental stresses.

Keywords: Booklice, *Liposcelis brunnea*, genome assembly, louse evolution, insecticide resistance, high temperature tolerance

1. Introduction

Psocids are stored-product arthropods that are of increasing economic importance as pests of seeds, raw agricultural materials, food and feed [1–4]. Booklice in the genus *Lipocelis* are the most important clade across the psocids because of their global distribution and high resistance to insecticides and fumigants [5]. More generally, booklice were known as minute, pale insects found scuttling across books or stacks of papers [6]. Booklice infestations are usually a result of poor storage conditions associated with high moisture, which negatively influences the commodity [7]. Psocids feeding can cause a 5-10% weight loss in agricultural commodities [8,9]. They can also have negative impacts on human health through the production of allergens [10,11] or transmission of parasites [12].

Contact insecticides and fumigants are used for managing booklice. However, booklice can develop insecticide resistance compared with other stored product pests [2]. For example, deltamethrin, carbaryl, and methoprene, which can control beetles and moths, are not effective against booklice [13,14]. Moreover, booklice are documented to possess high resistance to bacterium-derived spinosad, imidacloprid and diatomaceous earth [15,16]. Phosphine fumigation is the most popular method for managing storage pests [17]. A high level of resistance to phosphine has been observed in booklice, particularly during the egg stage, which significantly increases both the economic cost of treatments and environmental pollution [18,19]. Several gene families have been proven to be related to the high insecticide resistance of booklice, including esterases (EST) [20], glutathione S-transferase (GST) [21], and the cytochrome P450 monooxygenases (P450) [22]. These studies were mainly based on transcriptomic analyses and lacked whole genome data. We know that in certain circumstances, mRNA analysis can be influenced by insect age or other factors. These factors

can also affect gene family evaluation by eliminating those genes with low expression [23]. As a result, it is necessary to develop a nuclear genome of booklice species for resistance-related analyses.

Booklice are the phylogenetic sister group to parasitic lice and they have been considered a key taxon in determining the origins and evolution of parasitic lice [24–26]. The habits of *Liposcelis* species are similar to those of parasitic lice; for example, they are found in the nests of birds and mammals, indicating a close relationship with their potential host [27–29]. Therefore, identifying the shared features of booklice and parasitic lice, particularly their genome features, could provide unique insight into the origin of parasitism. To date, only two parasitic genomes have been published, and they present conflicting results with regard to the detoxification- and sensing-related gene families [30,31]. Uncovering a booklice genome, thus, could help resolve related questions.

Here, we present a high-quality genome assembly of *L. brunnea* (NCBI:txid209926) (Fig. 1), the first chromosome-level genome assembly reported in Psocoptera. PacBio sequencing, Illumina and HiC technology were leveraged in our study. Comparative genomics analysis provided new clues on the evolution of lice, and transcriptomic analysis revealed how booklice adapt to high temperature and insecticide treatment.

2. Methods

2.1 Sample collection

Samples of *L. brunnea* were collected in 2009 from Oklahoma State, in the United States and maintained for more than 100 breeding generations. We found a long and curly *Se* in

these samples, which is the typical morphological trait of *L. brunnea* compared with other booklice. The booklice were put in jars and fed a mixture of wheat flour, yeast and whole milk powder in a ratio of 10:1:1. The rearing jars were put into incubators in a dark environment with a temperature of 25 °C and a relative humidity of 75%.

2.2 DNA extraction, RNA extraction, library construction, and sequencing

We knew that the close relatives of booklice possessed a sex determination of XO [32], which indicated that we could assemble the complete set of chromosomes using either males or females. Genomic DNA of 500 adult females was extracted using a Promega Genomic DNA Purification Kit (A1125). After the quality of the isolated DNA was assessed, a ~20 kb library was constructed using a SMRTbell Express Template Prep Kit 2.0 (Pacific Biosciences, CA, USA). The library construction included DNA shearing, damage repair, end repair, hairpin adapter ligation, and purification. After a quality control test, the SMRTbell library was obtained. The library was sequenced using a single 8 M SMRT Cell on the PacBio Sequel II platform (PacBio Sequel II System, RRID:SCR_017990). For genome survey and assembly polishing, we extracted genomic DNA from 40 adult females and constructed an Illumina sequencing library according to the manufacturer's instructions (Illumina), which was then sequenced on the Illumina NovaSeq 6000 platform (Illumina NovaSeq 6000 Sequencing System, RRID:SCR_016387) in paired-end 150 bp mode (insert size 350 bp) for approximately 20 GB data. For genome annotation, we extracted total RNA from 40 adult females using the Tiangen RNA extraction kit. After reverse transcribing mRNA into cDNA, another Illumina library was constructed and sequenced with the same parameters for approximately 6 GB data. The sequencing processes were conducted by the Berry Genomics Company.

2.3 HiC sequencing

Approximately 500 adult females were collected for the HiC experiments and subsequent sequencing. The library was constructed using the following steps: crosslinking the crashed samples with formaldehyde, digesting the DNA with MboI enzyme, filling ends and marks with biotin, ligating the resulting blunt-end fragments, purification and random shearing of DNA into 300-500 bp fragments. After library construction following the manufacturer's instructions (Illumina), sequencing was performed on the Illumina NovaSeq 6000 platform in 150 bp paired-end mode for about 60 GB data. The experiments and sequencing were performed by Annoroad Gene Technology.

2.4 Genome survey, assembly, quality assessment

Using the Illumina sequencing reads, we counted the 19-mers with Jellyfish v2.2.10 (Jellyfish, RRID:SCR_005491) [33] and evaluated the genome features using GenomeScope v2.0 (GenomeScope, RRID:SCR_017014) [34]. The PacBio CLR data were processed using Canu v2.1.1 (Canu, RRID:SCR_015880) [35] following correction (-correct), trimming (-trim) and assembly (-assemble) with the following parameters: minReadLength=2000, minOverlapLength=500, corOutCoverage=120, corMinCoverage=2, correctedErrorRate=0.035. PacBio sequencing data and Illumina sequencing data were both leveraged to polish the draft genome. The PacBio sequencing data were mapped to the draft genome using pbmm2 v1.4.0 [36], after which gcpp v1.9.0 [37] with the arrow algorithm was used for assembly polishing. We then mapped the Illumina sequencing data to the gcpp-polished assembly using BWA v0.7.17 (BWA, RRID:SCR_010910) [38], and Pilon v1.23 (Pilon, RRID:SCR_014731) [39] was used to polish the second round. Because we set

“correctedErrorRate” to a very low level in the Canu assembly step, the heterogeneous contigs were separated, producing redundant contigs. After assembly polishing, purge_dups v1.2.5 (purge dups, RRID:SCR_021173) [40] was used for the redundancy purge. The filtered Hi-C reads were aligned to the polished genome by BWA v0.7.17 which was integrated into Juicer v1.6 (Juicer, RRID:SCR_017226) [41]. Only uniquely mapped and valid paired-end reads were used for assembly by 3D-DNA v180114 [42]. Juicebox v1.11.08 (Juicebox, RRID:SCR_021172) [43] was used to manually order the scaffolds to obtained the final chromosome assembly. BUSCO v5.1.3 (BUSCO, RRID:SCR_015008) [44] was used to assess the completeness of the genome assembly based on the arthropoda_odb10 database.

2.5 Genome annotation

RepeatModeler v2.0.1 (RepeatModeler, RRID:SCR_015027) [45] was used to build a custom *de novo* repeat library, based on which RepeatMasker v4.1.0 (RepeatMasker, RRID:SCR_012954) [46] was used to detect the repetitive elements. Genome structural annotation was conducted using the Maker pipeline v3.01.03 [47] with *ab initio* prediction, homology-based prediction and RNA-seq assisted prediction. The protein sequences from seven species (*Pediculus humanus*, *Frankliniella occidentalis*, *Tribolium castaneum*, *Drosophila melanogaster*, *Apis mellifera*, *Caenorhabditis elegans* and *Daphnia magna*) were fed to Maker for homology-based searches. The RNA-seq data were assembled using Trinity v2.11.0 (Trinity, RRID:SCR_013048) [48] software with the default parameters and the output transcripts were set as mRNA evidence. BLAST v2.10.0 (NCBI BLAST, RRID:SCR_004870) [49] and Exonerate v2.58.3 (Exonerate, RRID:SCR_016088) [50] were used to search and polish the homologous sequences. The first round output from the Maker analysis was collected and used to train gene models with SNAP v2006-07-28 (SNAP,

RRID:SCR_007936) [51] and Augustus v3.3.3 (Augustus, RRID:SCR_008417) [52]. Gene models from both software programs were fed into Maker for the second round run. Similarly, we ran a third round of gene model training and Maker prediction, after which we obtained the final version of the structural annotation results. Functional annotations were conducted on protein sequences using 1) DIAMOND BLASTP v2.0.14 [53] against the NCBI nr database; 2) InterProScan v1.8.0_312 (InterProScan, RRID:SCR_005829) [54] on Gene Ontology (GO) terms, Signal peptides (SignalP) and InterPro annotations; 3) eggNOG-mapper v2.1.7 (eggNOG-mapper, RRID:SCR_021165) [55] with COG category and KEGG pathways annotated.

2.6 Orthology prediction and phylogenetic analyses

Insects from Hemiptera (*Acyrthosiphon pisum* and *Bemisia tabaci*), Thysanoptera (*Frankliniella occidentalis* and *Thrips palmi*), Psocodea (*L. brunnea*, *Columbicola columbae* and *Pediculus humunus*) and Holometabola (*Drosophila melanogaster*, *Plutella xylostella* and *Tribolium castaneum*) were used in the orthology analysis with *Daphnia pulex* as the outgroup. Gene families including orthologous and paralogous gene families were detected by OrthoFinder v2.5.1 (OrthoFinder, RRID:SCR_017118) [56] using the default parameters. The protein sequences of all single copy genes were aligned using MAFFT v7.475 (MAFFT, RRID:SCR_011811) [57] and concatenated into a dataset. This dataset was used to construct a phylogenetic tree using FastTree v2.1.10 (FastTree, RRID:SCR_015501) [58]. MCMCTREE from PAML package v4.9 (PAML, RRID:SCR_014932) [59] was used to date this phylogenetic tree. We retrieved the divergence time between 1) *Drosophila melanogaster* and *Plutella xylostella* (243-317 MYA, million years ago) and 2) *Acyrthosiphon pisum*, *Bemisia tabaci* (158-351 MYA) from the TimeTree database [60].

2.7 Gene family expansion, contraction and annotation

The eleven species used in Section 2.6 (*Orthology prediction and phylogenetic analyses*) were selected to identify gene family expansion and contraction. CAFE v4.2.1 (CAFE, RRID:SCR_005983) [61], which leverages a birth and death rate model estimated over the inferred phylogeny, was used to compare gene family cluster expansion and contraction (-p 0.01). The gene family clusters were then annotated by selecting the dominant function across all their genes using KinFin v1.0 [62].

For each gene family, we manually annotated five insecticide resistance related gene families, ABC (ATP-binding cassette), EST, GST, P450 and UGT (UDP-glucuronosyl transferases); the HSP (heat shock protein) gene family; three sensing related gene families, CSP (chemosensory proteins), OBP (odourant‐ binding receptors) and OR (odourant receptors). The hidden Markov models (HMMs) of these gene families were downloaded from the Pfam database. The proteins of each gene family from *P. humanus*, *D. melanogaster* and *Bactrocera dorsalis* were downloaded. The HMMs and proteins were fed as the input for BLASTP v2.10.0 (BLASTP, RRID:SCR_001010) and HMMER v3.1b2 (Hmmer, RRID:SCR_005305) to search for related genes. BITACORA v1.3 [63] was used to incorporate both results in protein mode with an e-value of 1e-5. Protein sequences of the annotated P450 and HSP genes were aligned using MUSCLE v3.8.1551 (MUSCLE, RRID:SCR_011812) [64]. Both alignments were used to construct neighbour-joining trees using TreeBeST v1.9.2 (TreeBeST, RRID:SCR_018173) with 1,000 rounds of bootstrap testing. The trees were annotated and viewed using FigTree v1.4.2 (FigTree, RRID:SCR_008515).

2.8 Transcriptome analysis under phosphine fumigation/high temperature

We placed 40 adult females under phosphine (0.075 mg/L) for two hours as the insecticide treatment. High-temperature treatment employed 40 adult females, which were subjected to a temperature of 44 °C for two hours. After both treatments, total RNA was immediately extracted. The treatment and control groups were replicated four times with 12 transcriptomes sequenced. The RNA extraction and sequencing processes followed the methods described in Section 2.2 (*DNA extraction, RNA extraction, library construction, and sequencing*). After the quality control process, the sequencing data were mapped to the genome using HISAT2 v 2.2.1 (HISAT2, RRID:SCR_015530) [65] and quantified using FeatureCounts v2.0.1 (featureCounts, RRID:SCR_012919) [66]. Differentially expressed genes were analysed using edgeR v3.32.1 (edgeR, RRID:SCR_012802) [67].

3. Results

3.1 Genome sequencing and assembly

Altogether, 20.7 Gb of clean genome data (69,116,628 paired reads) were generated from the Illumina sequencing platform. The genome size was estimated to be 171.6 Mb with a heterogeneity of 0.268% (Fig. 2A). We obtained 52 Gb PacBio CLR data (2,733,343 subreads), which showed approximately 300-fold coverage with the subread N50 at 22.7 kb. After PacBio data correction, trimming and assembly by Canu, a draft genome was generated, including 2,071 contigs with a total size of 283.8 Mb and a contig N50 of 800 kb. The genome size was about 110 Mb larger than the surveyed genome, indicating that some heterogeneous contigs existed in this draft genome. The result of the BUSCO analysis also indicated the presence of redundant sequences, including 98.8% complete genes (C), of which 65.2% were single copy genes (S) and 33.6% were duplicated genes (D); 0.4% partial genes

(F) and 0.8% missed genes (M). We noticed good completeness but a high percentage of duplicated genes, which could be the result of redundant contigs. Insect species often possess a high heterogeneity that requires redundancy purging after the initial genome assembly [68]. After polishing the draft genome using PacBio and the Illumina sequencing data, purge_dups was used to purge the redundancy and produced a purged genome including 278 contigs, 178.9 Mb in size with a contig N50 of 1.78 Mb. The size of the purged genome is quite similar to our survey estimation and was subsequently used for the following HiC analysis.

Approximately 65.2 Gb of HiC data (217,199,354 read pairs) were produced and used to construct a chromosome-level genome assembly. After mapping the data to the purged genome, 162,444,941 unique read pairs were retrieved including 150,305,169 valid interaction read pairs, which indicated good quality HiC data (Fig. 2B). After manual checking, we obtained a genome assembly with the longest 9 linkage groups (LGs) covering 174.1 Mb (97.3% of genome bases). These LGs ranged from 12.1 Mb to 27.6 Mb in length and reached a scaffold N50 of 19.7 Mb (Fig. 2C). The BUSCO evaluation result of the nine LGs was C:98.9% [S:98.0%, D:0.9%], F:0.5%, M:0.6%. Compared with the only two genomes available in Psocodea (*P. humanus* and *C. columbae*), *L. brunnea* had a moderate genome size (Table 1), but had the largest contig N50, scaffold N50 and the best completeness evaluation, which indicated a high-quality genome.

3.2 Genome annotations

The structural annotation diagnosed 27,716,126 bp repeated sequences, constituting 15.92% of the *L. brunnea* genome. Retroelements and DNA transposons accounted for 3.81% and 1.24% of the genome, respectively. Of the retroelements, 2.61% of the genome sequence was identified as long interspersed elements (LINEs), 1.18% as long terminal repeats (LTRs)

and 0.03% as short interspersed elements (SINEs). There were also rolling-circles (0.62%), satellites (0.04%), simple repeats (0.98%), low complexity (0.36%) and unclassified repeat sequences (8.87%). The content of repetitive elements typically correlates with genome size [69,70], whereas exceptions exist in many cases partially because of the purging of heterogeneous contigs, or the nature of specific organisms [68,71]. Compared against *P. humanus* and *C. columbae* (Table 1), *L. brunnea* had the largest fraction of repetitive elements but with an intermediate genome size. The reduced size of transposable elements is considered to be common in lice and thus could be one reason for the reduction in genome size [31,72]. Moreover, the reduction of certain gene families, such as those related to sensing, also accounts for the tightening of louse genomes [30].

After Maker gene annotation, 15,543 genes were annotated in the genome of *L. brunnea*. The BUSCO result of this gene set was C:97.2% [S:95.8%, D:1.4%], F:1.2%, M:1.6%, indicating good quality structural annotation. Among all 15,543 genes, 12,157 genes were annotated by the nr database; 10,724 genes were annotated by InterProScan, with confirmed GO, SignalP and InterPro terms; 10,097 genes were annotated by eggNOG-mapper, together with the COGs and KEGGs. *L. brunnea* had 4,770 and 2,181 more genes than *P. humanus* and *C. columbae*, respectively. Regardless, compared with the other seven insect genomes, *P. humanus* and *C. columbae* also had the smallest gene numbers, indicating a large number of gene reductions in parasitic lice.

3.3 Gene orthology analysis and phylogeny reconstruction

In total, 16,563 gene families were identified, of which 1,448 were single copy genes in the OrthoFinder analysis (Fig. 3A). For *L. brunnea*, we assigned 12,530 genes to 9,144 gene families with 813 species-specific genes. For these insects, the unique genes ranged from 47

to 3,117, representing their specific evolutionary pathways, which will be explained in detail using gene family analysis.

Based on the phylogenetic reconstruction using single-copy genes, all species of Paraneoptera (Psocodea, Hemiptera and Thysanoptera) formed a clade, whereas other insects clustered together. However, several recent studies [26] reported that Psocodea might cluster with Holometabola insects but not with Hemiptera + Thysanoptera insects, which could be caused by unbalanced sampling and the different gene datasets used for phylogenetic inference. Our results thus indicated a closer relationship of the Paraneoptera clades. The MCMCTREE result indicated a divergence time between booklice and parasitic lice at ~231 MYA, which is similar to the results of several previous studies [25].

3.4 Gene family expansion and contraction

We first detected how the gene family evolved in Psocodea using CAFE, which might account for the formation of parasitism (Fig. 3). For the MRCA of booklouse (*L. brunnea*) and parasitic lice (*P. humanus* and *C. columbae*), 145 gene families expanded, whereas 3,757 contracted. The large number of contracted gene families indicates potential biological functional loss (Kirkness et al. 2010). Indeed, gene families including P450s, G protein-coupled receptors (GPCRs), OR, gustatory receptor (Gr) and CSP were contracted in both *P. humanus* and *C. columbae* (Table S1). Similar gene family (GPCRs and P450s) contraction was observed in the MRCA of booklice and parasitic lice, whereas there was a gene family expansion of Gr genes and no change in OR and CSP genes. These results indicate that the sense-related gene family changed mainly in the parasitic lice but not in the booklice. Conversely, the gene families of GPCR, OR, CSP and P450 expanded significantly in *L.*

brunnea, which might be explained by the requirements of a free-living lifestyle and adaptation to environmental change [73,74].

The BITOCORA analyses (Table 2) confirmed the Kinfin results with the three sensing related gene families (CSP, OR and OBP) contracted in parasitic lice and expanded in *L. brunnea*. Surprisingly, although parasitic lice and booklice live in different temperature conditions, they still have similar HSP gene numbers (~40). Moreover, we found that all insecticide resistance-related gene families (ABC, EST, GST, UGT and P450) kept their numbers in *L. brunnea* but were contracted in the two parasitic lice, indicating less environmental challenge to the latter clade. [75,76].

3.5 P450 genes in phosphine resistance

Among all insecticide resistance related gene families, we noticed that the P450 gene family was very large, with 125 P450 genes (Table 2). Regardless, *L. brunnea* has a large P450 gene family compared with all other closely related species. Four P450 subfamilies (CYP2, CYP3, CYP4 and Mito) of *L. brunnea* included respectively 13, 44, 50, 16 genes, whereas *F. occidentalis* had 10, 29, 43, 10 genes and *P. humunus* had 7, 12, 11, 10 genes for each subfamily (Fig. 4A). Compared with the parasitic lice, all four subfamilies of *L. brunnea* expanded significantly. The CYP4 subfamily had the largest number of genes, which could be the potential reason for high insecticide resistance. Similar CYP4 subfamily expansion was observed in *Thrips palmi*, which partially accounted for its high insecticide resistance [77].

We then analysed how P450 genes reacted to phosphine fumigation. After RNA-seq analyses, under the criteria of P value <0.05 and mRNA expression fold change >2, we found 11 differentially expressed genes (DEGs) from the P450 gene family (Fig. 4B, Table S2), distributed in the CYP4 (9 genes) and CYP2 (2 genes) subfamilies. Two DEGs were from the

CYP2 subfamily with one upregulated and the other downregulated. As predicted, most DEGs were from the CYP4 subfamily, seven of which were upregulated, suggesting that the largest P450 subfamily (CYP4) had the most important biological function with regard to phosphine resistance. Our results indicated that the high fumigant resistance in booklice species might originate from the expansion of the P450 gene family, particularly its CYP4 subfamily.

3.6 HSP genes in heat tolerance

Based on the fact that booklice favour high temperatures and HSP genes function during heat treatment across many species [78], we hypothesized that the free-living booklice possess an expanded HSP gene family. However, our gene family analyses proved that the HSP gene family of *L. brunnea* had a small number of genes across all species (Table 2). All three lice had approximately 40 P450 genes, indicating a similar evolutionary pathway in Psocodea (Psocoptera+ Phthiraptera). There could be two reasons for the conservation of HSP genes: 1) as an epibiont, parasitic lice still suffer fluctuating temperatures under various host activities; or 2) HSP genes are key components of other necessary biological functions, i.e., insect sleep [79], and thus are not influenced only by temperature conditions.

Five HSP subfamilies were identified in *L. brunnea* (Fig. 5A), including HSP20 (5), HSP40 (8), HSP60 (11), HSP70 (15) and HSP90 (6). After RNA-seq analysis, we found that 8 HSP genes from 4 subfamilies were upregulated significantly, indicating the importance of HSP genes for heat adaptation in booklice (Fig. 5B). HSP genes have been proven to be key in temperature adaptation in insects [80]. Our findings confirm these results and provide further evidence for how psocids have adapted to this important ecological aspect.

4. Conclusions

We report a high-quality genome assembly of *L. brunnea*, the type species in the genus *Liposcelis*. This is the first genome from the order Psocoptera uncovered. The genome of *L. brunnea* has a contig N50 of 1.78 Mb and is distributed into nine LGs. The lice clade, including booklice, barklice and parasitic lice, diversified for approximately 231 million years with sensing- and insecticide resistance-related gene families contracted in the latter clade. We found that P450 genes, particularly those from the CYP4 subfamily, affect phosphine fumigation and thus are key potential targets for genetic-based pest control methods. Approximately one-quarter of the HSP genes were upregulated under heat treatment, indicating their importance in temperature adaptation. Overall, our study provides valuable data and insights into lice evolution and environmental stress adaptation.

Data Accessibility

Illumina DNA/RNA sequencing data, PacBio sequel II genome sequencing and Hi-C data were uploaded at NCBI SRA under BioProject: PRJNA772023. The genome assembly is under NCBI WGS Accession: JAJEOV000000000. All other supporting data and materials are available in the *GigaScience* GigaDB database [81]

Additional Files

Table S1. Annotation of clade-specific expansion/contraction gene families from KinFin analysis.

Table S2. Differential expressed genes in P450 gene family during phosphine fumigation.

Competing Interests

The authors declare that they have no competing interests.

Funding

This work was supported by the Key Research Program of International Collaboration between China and Czech Republic (2018YFE0108700) to Z. L., China Agriculture Research System of MOF and MARA to Z. L.

Authors' Contributions

SF and ZL conceived the project and wrote the manuscript. GO, VS and ZL collected and identified the samples. SF performed the analyses. SF and WD performed the experiments. All authors read and approved the final manuscript.

Acknowledgements

We thank Yueyang Zhou, Dr Qianqian Yang for their help on sample rearing and data analysis.

References

1. Phillips TW, Throne JE. Biorational approaches to managing stored-product insects. *Annu Rev Entomol*. 2010; doi: 10.1146/annurev.ento.54.110807.090451.

2. Nayak MK, Collins PJ, Throne JE, Wang J. Biology and management of psocids infesting stored products. *Annu Rev Entomol*. 2014; doi: 10.1146/annurev-ento-011613-161947.

3. Stejskal V, Hubert J, Aulicky R, Kucerova Z. Overview of present and past and pest-associated risks in stored food and feed products: European perspective. *J Stored Prod Res*. 2015; doi: 10.1016/j.jspr.2014.12.006.

4. Athanassiou CG, Rumbos CI. Emerging Pests in Durable Stored Products. In: Athanassiou CG, Arthur

FH, editors. *Recent Advances in Stored Product Protection*. Berlin, Heidelberg: Springer Berlin Heidelberg; 2018.

5. Lienhard C, Smithers CN. Psocoptera (Insecta): World catalogue and bibliography. In: Lienhard C, Smithers CN, editors. Switzerland, Geneva: Muséum d'Histoire Naturelle de Genève.; 2002.

6. Grimaldi D, Engel MS. Fossil Liposcelididae and the lice ages (Insecta: Psocodea). *Proc Biol Sci*. 2006; doi: 10.1098/rspb.2005.3337.

7. Turner BD. Forming a clearer view of *L. bostrychophilus*. *Environ Health*. 1987; 95:9–13.

8. Macfarlane JA. Damage to milled rice by psocids. *Trop. Stored Prod. Inf.* 1982; 44:3-10.

9. Kučerová Z. Weight losses of wheat grains caused by psocid infestation. *Plant Prot Sci*. 2002; doi: 10.17221/4858-PPS.

10. Turner BD, Staines NA, Brostoff J, Howe CA, Cooper K, Wildey KB. Allergy to psocids. *Proceedings of the International Conference on Insect Pests in the Urban Environment (ICIPUE)*. ICIPUE, Heriot-Watt University Edinburgh, Scotland; p. 7–10.

11. Hubert J, Stejskal V, Athanassiou CG, Throne JE. Health Hazards Associated with Arthropod Infestation of Stored Products. *Annu Rev Entomol*. 2018; doi: 10.1146/annurev-ento-020117-043218.

12. Turner BD. *Liposcelis bostrychophila* (Psocoptera: Liposcelididae), a stored food pest in the UK. *Int J Pest Manage*. 1994; doi: 10.1080/09670879409371879.

13. Nayak MK, Collins PJ, Reid SR. Efficacy of Grain Protectants and Phosphine Against *Liposcelis bostrychophila*, *L. entomophila*, and *L. paeta* (Psocoptera: Liposcelidae). *J Econ*. 1998; doi: 10.1093/jee/91.5.1208.

14. Daglish GJ, Wallbank BE, Nayak MK. Synergized bifenthrin plus chlorpyrifos-methyl for control of beetles and psocids in sorghum in Australia. *J Econ Entomol*. 2003; doi: 10.1093/jee/96.2.525.

15. Nayak MK, Daglish GJ. Potential of imidacloprid to control four species of psocids (Psocoptera: Liposcelididae) infesting stored grain. *Pest Manag Sci*. 2006; doi: 10.1002/ps.1220.

16. Athanassiou CG, Arthur FH, Opit GP, Throne JE. Insecticidal effect of diatomaceous earth against three species of stored-product psocids on maize, rice, and wheat. *J Econ Entomol*. 2009; doi: 10.1603/029.102.0435.

17. Pike V. Laboratory assessment of the efficacy of phosphine and methyl bromide fumigation against all life stages of *Liposcelis entomophilus* (Enderlein). *Crop Prot*. Elsevier; 1994; doi: 10.1016/0261-2194(94)90165-1.

18. Cao Y, Song Y, Sun GY. A survey of psocid species infesting stored grain in China and resistance to phosphine in field populations of *Liposcelis entomophila* (Enderlein)(Psocoptera: Liposcelididae). *Proceedings of the 8th International Working Conference* 2003; 662-667.

19. Nayak MK, Collins PJ, Pavic H, Kopittke RA. Inhibition of egg development by phosphine in the cosmopolitan pest of stored products *Liposcelis bostrychophila* (Psocoptera: Liposcelididae). *Pest Manag Sci*. 2003; doi: 10.1002/ps.753.

20. Wei D, He W, Miao Z, Tu Y, Wang L, Dou W, et al.. Characterization of Esterase Genes Involving Malathion Detoxification and Establishment of an RNA Interference Method in *Liposcelis bostrychophila*. *Front Physiol*. 2020; doi: 10.3389/fphys.2020.00274.

21. Wu S, Dou W, Wu J, Wang J. Purification and partial characterization of glutathione S-transferase from

insecticide-resistant field populations of *Liposcelis paeta* Pearman (Psocoptera: Liposcelididae). *Arch Insect Biochem Physiol*. 2009; doi: 10.1002/arch.20285.

22. Wei D, Li T, Chen S, Dou W, Wang J. Molecular studies of psocids in China: recent advances. *proceedings of the 11th International Working Conference on Stored Product Protection* 2014; doi: 10.14455/DOA.res.2014.17.

23. Boivin V, Reulet G, Boisvert O, Couture S, Elela SA, Scott MS. Reducing the structure bias of RNA-Seq reveals a large number of non-annotated non-coding RNA. *Nucleic Acids Res*. 2020; doi: 10.1093/nar/gkaa028.

24. Lyal CHC. Phylogeny and classification of the Psocodea, with particular reference to the lice (Psocodea: Phthiraptera). *Syst Entomol*. 1985; doi: 10.1111/j.1365-3113.1985.tb00525.x.

25. Johnson KP, Dietrich CH, Friedrich F, Beutel RG, Wipfler B, Peters RS, et al. Phylogenomics and the evolution of hemipteroid insects. *Proc Natl Acad Sci USA*. 2018; doi: 10.1073/pnas.1815820115.

26. de Moya RS, Yoshizawa K, Walden KKO, Sweet AD, Dietrich CH, Johnson KP. Phylogenomics of parasitic and non-parasitic lice (Insecta: Psocodea): Combining sequence data and Exploring compositional bias solutions in Next Generation Datasets. *Syst Biol*. 2020; doi: 10.1093/sysbio/syaa075.

27. Broadhead E. A revision of the genus *Liposcelis* Motschulsky with notes on the position of this genus in the order Corrodentia and on the variability of ten *Liposcelis* species. *Transactions of the Royal Entomological Society of London*. 1950; doi: 10.1111/j.1365-2311.1950.tb00449.x.

28. Mockford EL. Psocoptera from sleeping nests of the dusky-footed wood rat in southern California (Psocoptera: Atropidae, Psoquillidae, Liposcelidae). *Pan pacific Entomol*. 1971; 47: 127-40

29. Baz A, Others. Psocoptera from weaver bird nests (Aves: Ploceidae) in Equatorial Guinea (West-Africa). *Annales de la Société entomologique de France*. 1990; 26: 33–8.

30. Kirkness EF, Haas BJ, Sun W, Braig HR, Perotti MA, Clark JM, et al. Genome sequences of the human body louse and its primary endosymbiont provide insights into the permanent parasitic lifestyle. *Proc Natl Acad Sci U S A*. 2010; doi: 10.1073/pnas.1003379107.

31. Baldwin-Brown JG, Villa SM, Vickrey AI, Johnson KP, Bush SE, Clayton DH, et al. The assembled and annotated genome of the pigeon louse *Columbicola columbae*, a model ectoparasite. *G3*. 2021; doi: 10.1093/g3journal/jkab009.

32. Wong SK, Thornton IW. Chromosome numbers of some psocid genera (Psocoptera). *Nature*. 1966; doi: 10.1038/211214a0.

33. Marçais G, Kingsford C. A fast, lock-free approach for efficient parallel counting of occurrences of k-mers. *Bioinformatics*. 2011; doi: 10.1093/bioinformatics/btr011.

34. Ranallo-Benavidez TR, Jaron KS, Schatz MC. GenomeScope 2.0 and Smudgeplot for reference-free profiling of polyploid genomes. *Nat Commun*. 2020; doi: 10.1038/s41467-020-14998-3.

35. Koren S, Walenz BP, Berlin K, Miller JR, Bergman NH, Phillippy AM. Canu: scalable and accurate long-read assembly via adaptive k-mer weighting and repeat separation. *Genome Res*. 2017; doi: 10.1101/gr.215087.116.

36. Pacific Biosciences. pbmm2. https://github.com/PacificBiosciences/pbmm2. Accessed 26 May 2022

37. Pacific Biosciences. gcpp. https://github.com/PacificBiosciences/gcpp. Accessed 26 May 2022

38. Li H. Aligning sequence reads, clone sequences and assembly contigs with BWA-MEM. *arXiv*. 2013:

1303.3997v2.

39. Walker BJ, Abeel T, Shea T, Priest M, Abouelliel A, Sakthikumar S, et al. Pilon: an integrated tool for comprehensive microbial variant detection and genome assembly improvement. *PLoS One*. 2014; doi: 10.1371/journal.pone.0112963.

40. Guan D, McCarthy SA, Wood J, Howe K, Wang Y, Durbin R. Identifying and removing haplotypic duplication in primary genome assemblies. *Bioinformatics*. 2020; doi: 10.1093/bioinformatics/btaa025.

41. Durand NC, Shamim MS, Machol I, Rao SSP, Huntley MH, Lander ES, et al. Juicer provides a one-click system for analyzing loop-resolution Hi-C experiments. *Cell Syst*. 2016; doi: 10.1016/j.cels.2016.07.002.

42. Dudchenko O, Batra SS, Omer AD, Nyquist SK, Hoeger M, Durand NC, et al. De novo assembly of the *Aedes aegypti* genome using Hi-C yields chromosome-length scaffolds. *Science*. 2017; doi: 10.1126/science.aal3327.

43. Durand NC, Robinson JT, Shamim MS, Machol I, Mesirov JP, Lander ES, et al. Juicebox provides a visualization system for Hi-C contact maps with unlimited zoom. *Cell Syst*. 2016; doi: 10.1016/j.cels.2015.07.012.

44. Seppey M, Manni M, Zdobnov EM. BUSCO: Assessing genome assembly and annotation completeness. *Methods Mol Biol*. 2019; doi: 10.1007/978-1-4939-9173-0_14.

45. Flynn JM, Hubley R, Goubert C, Rosen J, Clark AG, Feschotte C, et al. RepeatModeler2 for automated genomic discovery of transposable element families. *Proc Natl Acad Sci U S A*. 2020; doi: 10.1073/pnas.1921046117.

46. Chen N. Using RepeatMasker to identify repetitive elements in genomic sequences. *Curr Protoc Bioinformatics*. 2004; doi: 10.1002/0471250953.bi0410s05.

47. Campbell MS, Holt C, Moore B. Genome annotation and curation using MAKER and MAKER-P. *Curr Protoc Bioinformatics.* 2014; doi: 10.1002/0471250953.bi0411s48.

48. Grabherr MG, Haas BJ, Yassour M, Levin JZ, Thompson DA, Amit I, et al.. Full-length transcriptome assembly from RNA-Seq data without a reference genome. *Nat Biotechnol*. nature.com; 2011; doi: 10.1038/nbt.1883.

49. Camacho C, Coulouris G, Avagyan V, Ma N, Papadopoulos J, Bealer K, et al.. BLAST+: architecture and applications. *BMC Bioinformatics*. Springer; 2009; doi: 10.1186/1471-2105-10-421.

50. Slater GSC, Birney E. Automated generation of heuristics for biological sequence comparison. *BMC Bioinformatics*. 2005; doi: 10.1186/1471-2105-6-31.

51. Korf I. Gene finding in novel genomes. *BMC Bioinformatics*. 2004; doi: 10.1186/1471-2105-5-59.

52. Stanke M, Morgenstern B. AUGUSTUS: a web server for gene prediction in eukaryotes that allows user-defined constraints. *Nucleic Acids Res*. 2005; doi: 10.1093/nar/gki458.

53. Buchfink B, Reuter K, Drost H-G. Sensitive protein alignments at tree-of-life scale using DIAMOND. *Nat Methods*. 2021; doi: 10.1038/s41592-021-01101-x.

54. Zdobnov EM, Apweiler R. InterProScan–an integration platform for the signature-recognition methods in InterPro. *Bioinformatics*. 2001; doi: 10.1093/bioinformatics/17.9.847.

55. Huerta-Cepas J, Forslund K, Coelho LP, Szklarczyk D, Jensen LJ, von Mering C, et al.. Fast genome-wide functional annotation through orthology assignment by eggNOG-Mapper. *Mol Biol Evol*. 2017; doi:

10.1093/molbev/msx148.

56. Emms DM, Kelly S. OrthoFinder: phylogenetic orthology inference for comparative genomics. *Genome Biol*. 2019; doi: 10.1186/s13059-019-1832-y.

57. Katoh K, Standley DM. MAFFT multiple sequence alignment software version 7: improvements in performance and usability. *Mol Biol Evol*. 2013; doi: 10.1093/molbev/mst010.

58. Price MN, Dehal PS, Arkin AP. FastTree: computing large minimum evolution trees with profiles instead of a distance matrix. *Mol Biol Evol*. 2009; doi: 10.1093/molbev/msp077.

59. Yang Z. PAML 4: phylogenetic analysis by maximum likelihood. *Mol Biol Evol*. 2007; doi: 10.1093/molbev/msm088.

60. Hedges SB, Dudley J, Kumar S. TimeTree: a public knowledge-base of divergence times among organisms. *Bioinformatics*. 2006; doi: 10.1093/bioinformatics/btl505.

61. De Bie T, Cristianini N, Demuth JP, Hahn MW. CAFE: a computational tool for the study of gene family evolution. *Bioinformatics*. 2006; doi: 10.1093/bioinformatics/btl097.

62. Laetsch DR, Blaxter ML. KinFin: software for taxon-aware analysis of clustered protein sequences. *G3*. 2017; doi: 10.1534/g3.117.300233.

63. Vizueta J, Sánchez-Gracia A, Rozas J. bitacora: A comprehensive tool for the identification and annotation of gene families in genome assemblies. *Mol Ecol Resour*. 2020; doi: 10.1111/1755-0998.13202.

64. Edgar RC. MUSCLE: multiple sequence alignment with high accuracy and high throughput. *Nucleic Acids Res*. 2004; doi: 10.1093/nar/gkh340.

65. Kim D, Paggi JM, Park C, Bennett C, Salzberg SL. Graph-based genome alignment and genotyping with HISAT2 and HISAT-genotype. *Nat Biotechnol*. 2019; doi: 10.1038/s41587-019-0201-4.

66. Liao Y, Smyth GK, Shi W. featureCounts: an efficient general purpose program for assigning sequence reads to genomic features. *Bioinformatics*. 2014; doi: 10.1093/bioinformatics/btt656.

67. Robinson MD, McCarthy DJ, Smyth GK. edgeR: a Bioconductor package for differential expression analysis of digital gene expression data. *Bioinformatics*. 2010; doi: 10.1093/bioinformatics/btp616.

68. Li F, Zhao X, Li M, He K, Huang C, Zhou Y, et al. Insect genomes: progress and challenges. *Insect Mol Biol*. 2019; doi: 10.1111/imb.12599.

69. Kidwell MG. Transposable elements and the evolution of genome size in eukaryotes. *Genetica*. 2002; doi: 10.1023/a:1016072014259.

70. Haubold B, Wiehe T. How repetitive are genomes? *BMC Bioinformatics*. 2006; doi: 10.1186/1471-2105-7-541.

71. Dennis AB, Ballesteros GI, Robin S, Schrader L, Bast J, Berghöfer J, et al. Functional insights from the GC-poor genomes of two aphid parasitoids, *Aphidius ervi* and *Lysiphlebus fabarum*. *BMC Genomics*. 2020; doi: 10.1186/s12864-020-6764-0.

72. Lefébure T, Morvan C, Malard F, François C, Konecny-Dupré L, Guéguen L, et al. Less effective selection leads to larger genomes. *Genome Res*. 2017; doi: 10.1101/gr.212589.116.

73. Brand P, Ramírez SR. The evolutionary dynamics of the odorant receptor gene family in corbiculate bees. *Genome Biol Evol*. 2017; doi: 10.1093/gbe/evx149.

74. Ritschard EA, Fitak RR, Simakov O, Johnsen S. Genomic signatures of G-protein-coupled receptor

expansions reveal functional transitions in the evolution of cephalopod signal transduction. *Proc Biol Sci*. 2019; doi: 10.1098/rspb.2018.2929.

75. Sun G, Xu Y, Liu H, Sun T, Zhang J, Hettenhausen C, et al. Large-scale gene losses underlie the genome evolution of parasitic plant *Cuscuta australis*. *Nat Commun*. 2018; doi: 10.1038/s41467-018-04721-8.

76. ChuanLin Y, XinHai Y, MengYao C, Yang M, HuaMei X, Fei L. Evolution analysis of cytochrome P450 gene family in parasitoid wasps. *Zhongguo Sheng Wu Fang Zhi*. 2019; 35:335–42.

77. Guo S, Cao L, Song W, Shi P, Gao Y, Gong Y, et al. Chromosome-level assembly of the melon thrips genome yields insights into evolution of a sap-sucking lifestyle and pesticide resistance. *Mol Ecol Resour*. 2020; doi: 10.1111/1755-0998.13189.

78. Gu X, Zhao Y, Su Y, Wu J, Wang Z, Hu J, et al. A transcriptional and functional analysis of heat hardening in two invasive fruit fly species, *Bactrocera dorsalis* and *Bactrocera correcta*. *Evol Appl*. 2019; doi: 10.1111/eva.12793.

79. Zhao X, Li Y, Zhao Z, Du J. Extra sex combs buffers sleep-related stresses through regulating Heat shock proteins. *The FASEB Journal*. 2021; doi: 10.1096/fj.202001303RR.

80. González-Tokman D, Córdoba-Aguilar A, Dáttilo W, Lira-Noriega A, Sánchez-Guillén RA, Villalobos F. Insect responses to heat: physiological mechanisms, evolution and ecological implications in a warming world. *Biol Rev Camb Philos Soc*. 2020; doi: 10.1111/brv.12588.

81. Feng S; Opit G; Deng W; Stejskal V; Li Z. Supporting data for "A chromosome-level genome of the booklouse, *Liposcelis brunnea* provides insight into lice evolution and environmental stress adaptation" *GigaScience Database*.2022. http://doi.org/10.5524/102222.

Tables

Table 1. The genome features of *L. brunnea* and two parasitic lice.

Genome Features	Parasitic Lice		Booklice
	Pediculus humanus	*Columbicola columbae*	*Liposcelis brunnea*
Genome Size (MB)	110	208	174
Chromosomes	6	12	9
Methods	Capillary Platform	Nanopore+Illumina+HiC	PacBio+Illumina+HiC
Contig N50	-	511 kb	1.78 Mb
Scaffold N50	488 kb	17.6 Mb	19.7 Mb
Genes	10,773	13,362	15,543
Repetitive elements	7.3% (8.0 Mb)	9.7% (20.2 Mb)	15.9% (27.7 Mb)
BUSCO Evaluation	95.9%	96.4%	97.2%

Table 2. Statistics on detoxification, heat-shock protein (HSP) and sensing related genes across Psocodea insects and other insects.

Gene Family	Psocodea			Hemiptera			Thysanoptera		Diptera
	Pediculus humunus	*Columbicola columbae*	*Liposcelis brunnea*	*Acyrthosiphon pisum*	*Bemisia tabaci*	*Nilaparvata lugens*	*Frankliniella occidentalis*	*Thrips palmi*	*Drosophila melanogaster*
ABC	43	47	**66**	107	53	80	65	57	57
EST	26	29	**69**	45	49	81	66	78	42
GST	24	18	**44**	34	32	26	33	36	54
UGT	4	3	**19**	60	80	20	26	18	35
P450	43	44	**125**	79	141	88	95	115	92
HSP	41	39	**42**	48	44	71	71	63	58
CSP	6	8	**9**	10	19	17	11	11	4
OBP	3	5	**37**	18	8	23	16	24	47
OR	10	9	**29**	17	10	31	13	15	67

Figures

Figure 1. The adult booklouse, *Liposcelis brunnea* in the present study. They are reared in artificial diet in the lab (left figure). They are always trapped in the grain storage, especially the corner of storage facilities (right figures).

Figure 2. Genome survey, HiC and basic genomic features. (a) GenomeScope estimation of genome size, heterogeneity using a k-mer of 19. (b) HiC interaction map produced by 3D-DNA. (c) Circos plot of the linkage groups. The outer 9 bars represented 9 linkage groups while the red, green, blue heat maps represented gene counts, repeat sequences and GC content, respectively, in 50 kb windows.

Figure 3. Gene family evolution among *L. brunnea* and other insects. Phylogenetic trees inferred from concatenated single copy genes using Fasttree. MCMCTREE was used for molecular dating. The single copy genes, multiple copy genes, species specific genes, clade specific genes were analyzed based on the results from OrthoFinder. Gene family expansion (green) and contraction (red) was analyzed using CAFE.

Figure 4. Phylogenetic tree of P450 gene family and significantly expressed genes. (A) The P450 genes of *L. brunnea* (in black), *P. humanus* (in purple), *D. melanogaster* (in red) from BITACORA analysis were used to construct a neighbor-joining tree where four subfamilies separated obviously. (B) Totally four treatment (P1-P4) and four control (C1-C4) groups were analysed. The subfamilies of 11 differential expressed P450 genes were listed. The expression data was normalized for each gene.

Figure 5. Phylogenetic tree of HSP gene family and significantly expressed genes. (A) The HSP genes of *L. brunnea* (in black) and *D. melanogaster* (in red) from BITACORA analysis were used to construct a neighbor-joining tree where five subfamilies separated obviously. (B) Totally four treatment (T44_1-T44_4) and four control (T25_1-T25_4) groups were analysed. The subfamilies of 8 differential expressed HSP genes were listed. The expression data was normalized for each gene.

Figure1

Click here to access/download;Figure;Figure 1.tif

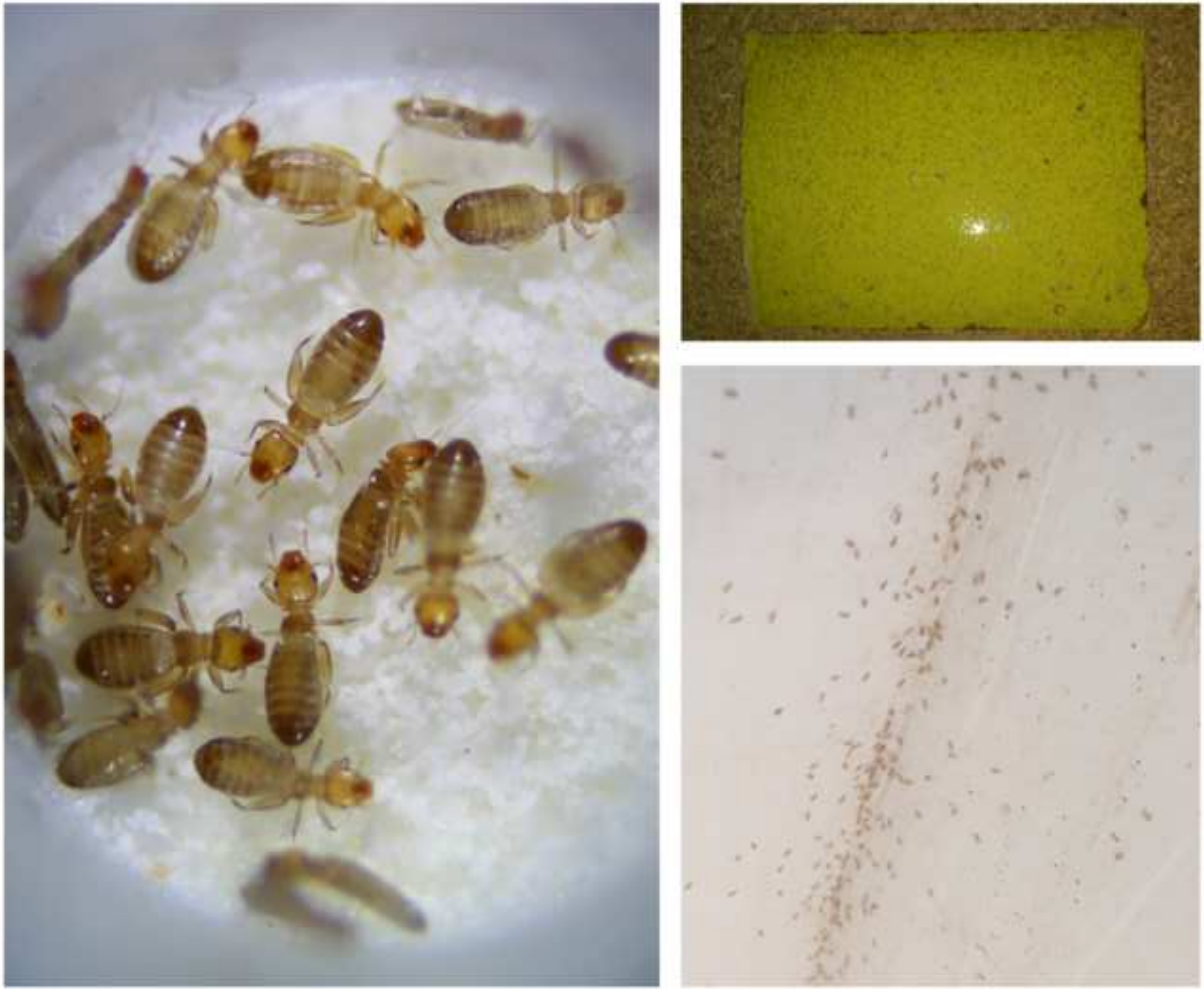

Figure2 Click here to access/download;Figure;Figure 2.tif

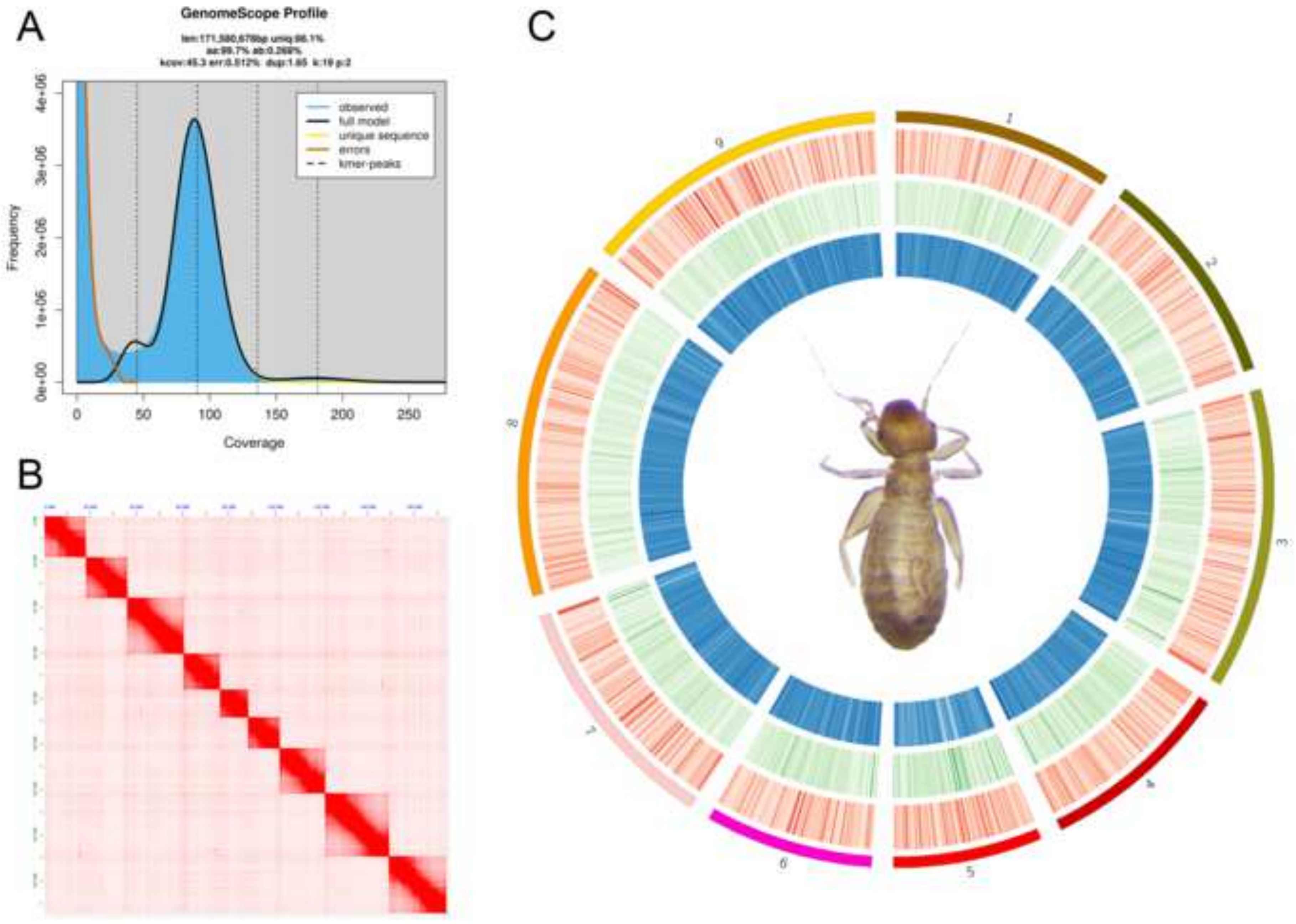

Figure3

Click here to access/download;Figure;Figure 3.tif

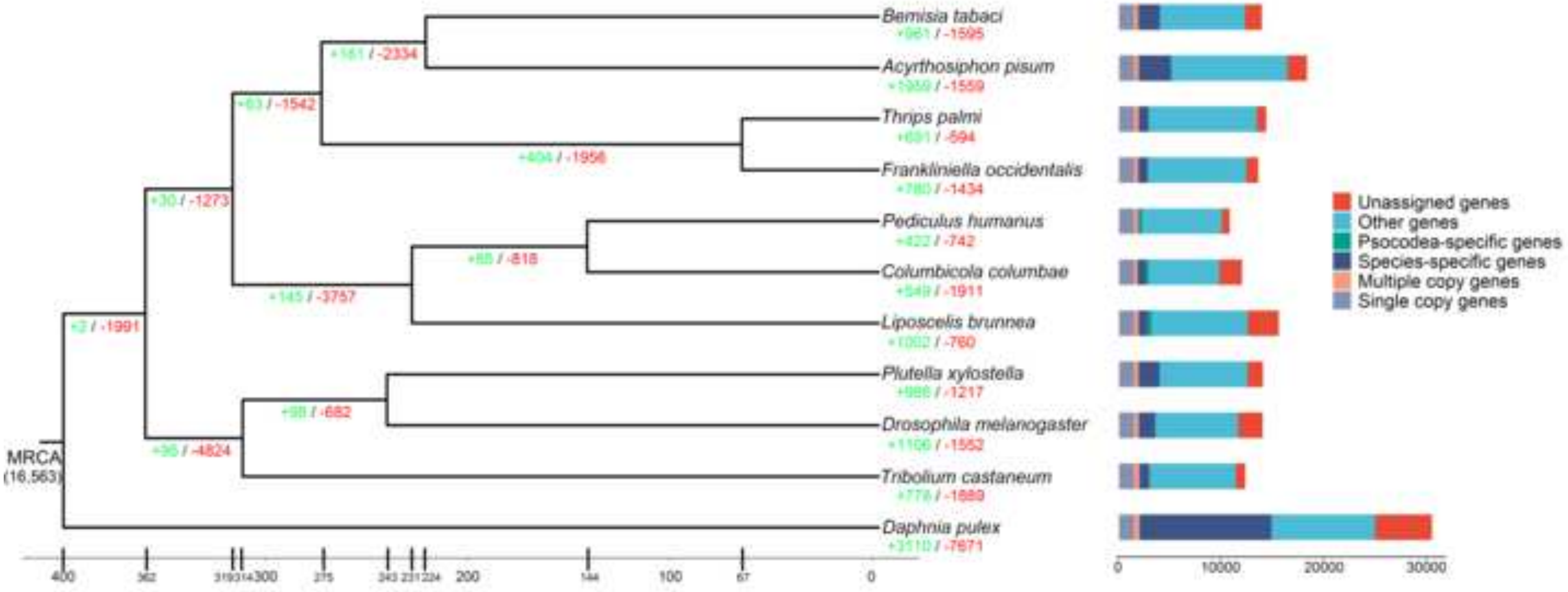

Figure4

Click here to access/download;Figure;Figure 4.tif

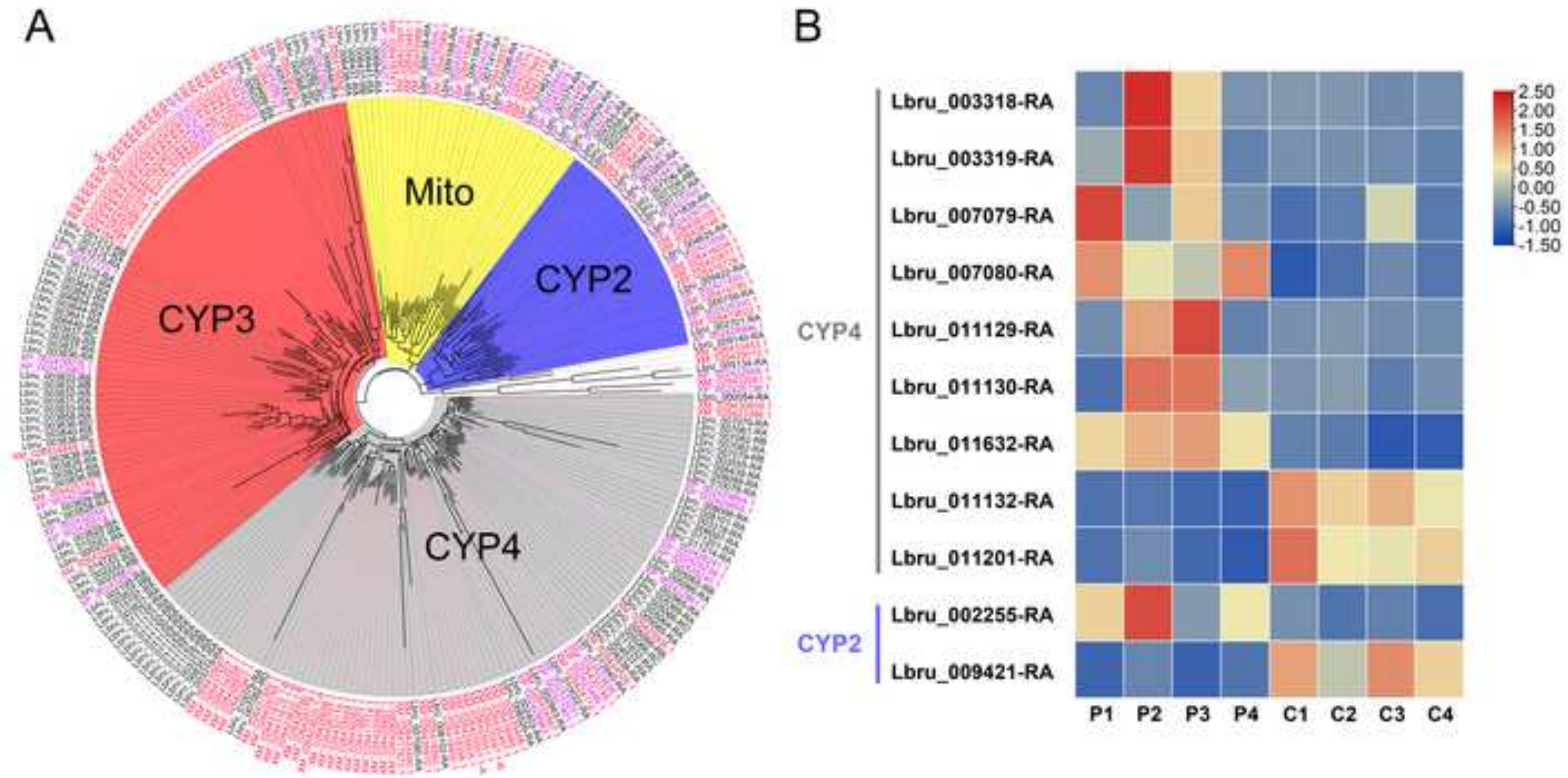

Figure5

Click here to access/download;Figure;Figure 5.tif

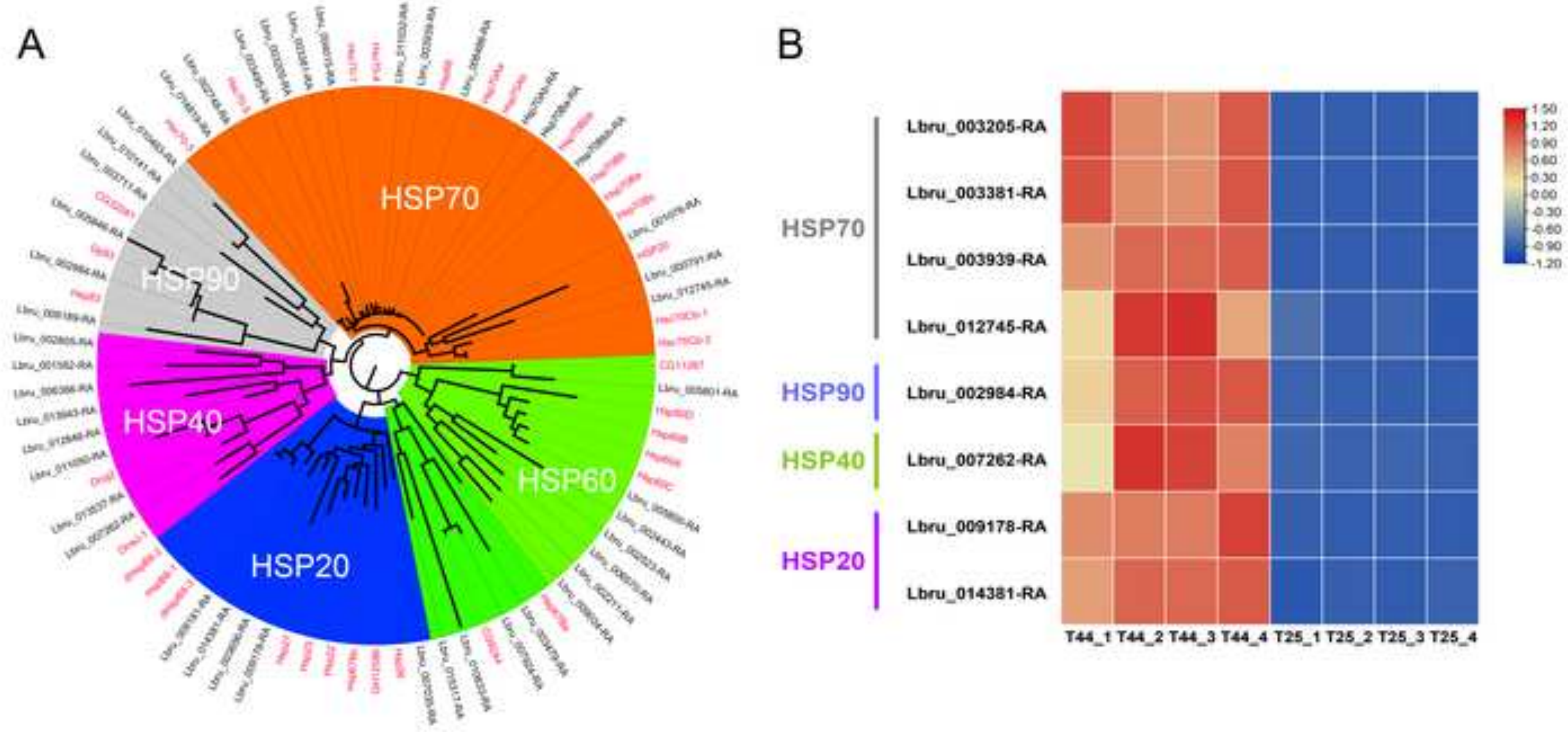

Manuscript with changes tracked

Click here to access/download
Supplementary Material
Main_Manuscript_changes_tracked.docx

Response letter

Click here to access/download
Supplementary Material
Response letter GIGA.docx

Language modification certificate

Click here to access/download
Supplementary Material
Language_modification_certificate_AJE.pdf

Table S1

Click here to access/download
Supplementary Material
Table S1.xlsx

Table S2

Click here to access/download
Supplementary Material
Table S2.xlsx
