# Supplementary material for: A chromosome-level genome of the booklouse, Liposcelis brunnea, provides insight into louse evolution and environmental stress adaptation
Source: Gigascience. 2022 Jul 19;11:giac062. doi: 10.1093/gigascience/giac062 (PMC9295366; doi:10.1093/gigascience/giac062)
Supplement: giac062_Response_to_Reviewer_Comments_Original_Submission [file giac062_response_to_reviewer_comments_original_submission.pdf]

Author's Response To Reviewer Comments

Close

We thank the editor's help for processing our manuscript. We thank two reviewers for their valuable comments on our work. We believe that through their comments, we were able to significantly improve our work. We have responded to each of the points made by the reviewers here. We hope you will find the revised version of the manuscript improved, and suitable for publication. We submitted the clean modified manuscript file and the file with changes tracked. We specified all the modifications in the response letter and please don't hesitate to contact us if there are any ambiguous points or files necessary for our manuscript evaluation.

Reviewer #1:
This manuscript by Feng et al. titled "A chromosome-level genome of the booklouse, Liposcelis brunnea provides insight into lice evolution and environmental stress adaptation" provides useful information on the genome organization, transcriptome, and some aspects of physiology. Authors have used technology typically used for sequencing, assembling, scaffolding, and annotating genomes and satisfactorily achieved their goals. The manuscript is sufficiently descriptive and easy to follow with sufficient technical details and references to appropriate manuscripts. They have provided parameters used in various analysis steps when deviated from defaults. Overall, the manuscript is in very good shape except for several sentence structure and English language issues which can be corrected easily with professional assistance.

Response: We thank the reviewer for their interest in our paper and the valuable insights. We have conducted language polish by all the authors and with professional assistance from American Journal Experts company.

Please see some of the issues I believe that needs to be fixed:
Line 24: The sentence starting with "Totally 15,543 genes..." may be revised to include written language.
Line 31: Conclusions: Insufficient details. Filling in the blanks? I think you can do a better job than this.
Line 49: Word "which" is duplicated.
Line 175: "For each gene family, we manually annotated five gene resistance related families..." Not sure what authors meant by "five gene resistance related families." First, the authors should define what type of resistance they are discussing here. Is it resistance to corrosion? I guess not. I am sure the authors are referring to insecticide resistance. Please revise the sentences to make sense and be specific.

Response: We modified "Totally" to "In total" (Line 25 in Main_Manuscript_changes_tracked.docx) and supplemented necessary content in the Abstract/Conclusions (Lines 33-37). The duplicated "which" was removed (Line 55) and the descriptive word "insecticide" was added before "resistance" (Line 204).

Table 1: Title needs to be more descriptive to indicate what is shown in this table.
Table 2: Title is too short and not descriptive enough to indicate what is shown in this table.

Response: We changed the titles of two tables (Lines 772, 774) and made them more descriptive.

Figure 2: Figures are too small. Hope the digital version is of high resolution and readable.
Figure 2 title: "...repeat sequences and GC content in 50 kb windows." should be "...repeat sequences and GC content, respectively, in 50 kb windows."
Figures 4A and 5A: Too small and crammed to be a useful figure. Needs to make it substantially large.
Above are only a representative sample of revisions required to the manuscript text to improve the readability.

Response: We attached the high-resolution figures of Figure 2, 4, 5 and we believe these figures would solve reviewer #1's figure-related questions. The title of Figure 2 was modified as suggested (Line 788). We thank reviewer #1 for the valuable and detailed advices.

Reviewer #2:
Booklice is a group of important storage pests that can live under heat treatment and usually possess high resistance to common fumigant insecticides. This paper reported the first genome in Psocoptera with high quality, using PacBio long-read sequencing, Illumina sequencing and HiC methods, and was well annotated. Gene family analyses and transcriptomic analysis provide new insights into lice evolution and the strategy of how insects adapt to environmental stresses. This study combines experimental biology evidence and provides valuable data to provide new insights for future studies. Here are my comments:

Response: We thank the reviewer for their interest and comment of our work.

Line 24, Totally, change to, in total
Line 190, ppm, change to, mg/L
Line 190, methods of treating the adults need to be described in detail. How long time did you treat the adults before the collection of samples?

Response: We modified the manuscript following the reviewer's suggestions (Lines 25, 220 in Main_Manuscript_changes_tracked.docx). The words were modified accordingly and the fumigation/high temperature treatments were detailly described (Lines 220-223).

In Samples collection, feeding conditions and feed ingredients are introduced. I suggest adding sampling time, breeding generation and identification method, which may help to explain the high heterozygosity of the draft genome. Furthermore, I hope the authors describe why only the females were selected for sequencing and transcriptome analysis.

Response: We added the information of sampling time, breeding generation and identification traits of Liposcelis brunnea (Lines 94-97). For the heterozygosity, the evaluated value is 0.268%, which should be relatively small across Insecta. We believe the redundant contigs during the assembly process is mainly from the parameters of Canu, where "correctedErrorRate=0.035" is a quite critical value to separate similar sequences, i.e., Canu is prone to keep the contigs even when they have only a few SNPs. This is also a try to see if we can assemble the diploid genome directly. However, nothing we could do with only these redundant contigs, so we chose to purge them as most researchers and studies have done. We also supplemented the reason why we use females (Lines 103-105), that is, L. brunnea might have a sex determination pattern of XO, meaning we can assemble the whole set of chromosomes using either females or males. Females have a large body size and more DNA, so we can use minimum insects for sequencing which also help eliminate the meaningless heterozygosity.

In the part of Methods, there is much software in this part which needs to be checked carefully. Some software lacks versions or literature information, such as "Juicer" in line 125 and "Juicebox" in 126 lack versions information, and in line 157 "Fasttree" lacks reference information.

Response: We followed the reviewer's suggestions and supplemented the version, references of the software. Moreover, we carefully checked all software in Methods part and added version and reference information.

In the part of Transcriptome analysis under phosphine fumigation/high temperature, there is no definite basis for phosphine concentration and temperature. This process may affect the number and type of differential expressed genes. I hope the author can explain why they choose this concentration and temperature.

Response: In this study, we would like to check the initial transcriptomic response of L. brunnea to the stresses, so we set the treatment time at 2 hours. There is no direct phosphine test of L. brunnea, thus we used the data from a close species, L. bostrychophila as the reference. Mangoba et al. (2021) found a complete control of L. bostrychophila at 0.1-0.5 mg/L for 72h. So we chose to take a less lethal concentration at 0.075 mg/L. For temperature related study, we want to have an extraordinarily high temperature treatment. Pre-experiments showed all L. brunnea individuals survived after 2h treatment under 44℃, which also satisfied our requirement of alive samples before RNA extraction.

Reference: Mangoba, M. A. A., & de Guzman Alvindia, D. (2021). Phosphine Resistance in Psocid, Liposcelis bostrychophila (Psocoptera) in the Philippines. International Journal of Tropical Insect Science,

41(1), 439–445.

Furthermore, we went through the manuscript once again to check and remove typos and correct spelling errors.

Response: We go over the manuscript once again and check the grammar and spellings. We also used a language modification service from the American Journal Experts to better standardize our manuscript. Thanks for the reviewer’s suggestions, which helped improve our manuscript greatly.

Close
