# Supplementary material for: A chromosome-level genome of the booklouse, Liposcelis brunnea, provides insight into louse evolution and environmental stress adaptation
Source: Gigascience. 2022 Jul 19;11:giac062. doi: 10.1093/gigascience/giac062 (PMC9295366; doi:10.1093/gigascience/giac062)
Supplement: giac062_Reviewer_1_Report_Original_Submission — Omaththage Perera -- 4/20/2022 Reviewed [file giac062_reviewer_1_report_original_submission.pdf]

Reviewer Report

Title: A chromosome-level genome of the booklouse, Liposcelis brunnea provides insight into louse evolution and environmental stress adaptation

Version: Original Submission **Date:** 4/20/2022

Reviewer name: Omaththage Perera

Reviewer Comments to Author:

This manuscript by Feng et al. titled "A chromosome-level genome of the booklouse, Liposcelis brunnea provides insight into lice evolution and environmental stress adaptation" provides useful information on the genome organization, transcriptome, and some aspects of physiology. Authors have used technology typically used for sequencing, assembling, scaffolding, and annotating genomes and satisfactorily achieved their goals. The manuscript is sufficiently descriptive and easy to follow with sufficient technical details and references to appropriate manuscripts. They have provided parameters used in various analysis steps when deviated from defaults. Overall, the manuscript is in very good shape except for several sentence structure and English language issues which can be corrected easily with professional assistance.

Please see some of the issues I believe that needs to be fixed:

Line 24: The sentence starting with "Totally 15,543 genes..." may be revised to include written language.

Line 31: Conclusions: Insufficient details. Filling in the blanks? I think you can do a better job than this.

Line 49: Word "which" is duplicated.

Line 175: "For each gene family, we manually annotated five gene resistance related families..." Not sure what authors meant by "five gene resistance related families." First, the authors should define what type of resistance they are discussing here. Is it resistance to corrosion? I guess not. I am sure the authors are referring to insecticide resistance. Please revise the sentences to make sense and be specific.

Table 1: Title needs to be more descriptive to indicate what is shown in this table.

Table 2: Title is too short and not descriptive enough to indicate what is shown in this table.

Figure 2: Figures are too small. Hope the digital version is of high resolution and readable.

Figure 2 title: "...repeat sequences and GC content in 50 kb windows." should be "...repeat sequences and GC content, respectively, in 50 kb windows."

Figures 4A and 5A: Too small and crammed to be a useful figure. Needs to make it substantially large.

Above are only a representative sample of revisions required to the manuscript text to improve the readability.

Level of Interest

Please indicate how interesting you found the manuscript: Choose an item.

Quality of Written English

Please indicate the quality of language in the manuscript: Choose an item.

Declaration of Competing Interests

Please complete a declaration of competing interests, considering the following questions:

- Have you in the past five years received reimbursements, fees, funding, or salary from an organisation that may in any way gain or lose financially from the publication of this manuscript, either now or in the future?
- Do you hold any stocks or shares in an organisation that may in any way gain or lose financially from the publication of this manuscript, either now or in the future?
- Do you hold or are you currently applying for any patents relating to the content of the manuscript?
- Have you received reimbursements, fees, funding, or salary from an organization that holds or has applied for patents relating to the content of the manuscript?
- Do you have any other financial competing interests?
- Do you have any non-financial competing interests in relation to this paper?

If you can answer no to all of the above, write 'I declare that I have no competing interests' below. If your reply is yes to any, please give details below.

I declare that I have no competing interests.

I agree to the open peer review policy of the journal. I understand that my name will be included on my report to the authors and, if the manuscript is accepted for publication, my named report including any attachments I upload will be posted on the website along with the authors' responses. I agree for my report to be made available under an Open Access Creative Commons CC-BY license (http://creativecommons.org/licenses/by/4.0/). I understand that any comments which I do not wish to be included in my named report can be included as confidential comments to the editors, which will not be published.

Choose an item.

To further support our reviewers, we have joined with Publons, where you can gain additional credit to further highlight your hard work (see: https://publons.com/journal/530/gigascience). On publication of this paper, your review will be automatically added to Publons, you can then choose whether or not to claim your Publons credit. I understand this statement.

Yes Choose an item.
