# Supplementary material for: A chromosome-level genome of the booklouse, Liposcelis brunnea, provides insight into louse evolution and environmental stress adaptation
Source: Gigascience. 2022 Jul 19;11:giac062. doi: 10.1093/gigascience/giac062 (PMC9295366; doi:10.1093/gigascience/giac062)
Supplement: giac062_Reviewer_2_Report_Original_Submission — Shu-Jun Wei -- 4/23/2022 Reviewed [file giac062_reviewer_2_report_original_submission.pdf]

Reviewer Report

Title: A chromosome-level genome of the booklouse, Liposcelis brunnea provides insight into louse evolution and environmental stress adaptation

Version: Original Submission **Date:** 4/23/2022

Reviewer name: Shu-Jun Wei

Reviewer Comments to Author:

Booklice is a group of important storage pests that can live under heat treatment and usually possess high resistance to common fumigant insecticides. This paper reported the first genome in Psocoptera with high quality, using PacBio long-read sequencing, Illumina sequencing and HiC methods, and was well annotated. Gene family analyses and transcriptomic analysis provide new insights into lice evolution and the strategy of how insects adapt to environmental stresses. This study combines experimental biology evidence and provides valuable data to provide new insights for future studies.
Here are my comments:
Line 24, Totally, change to, in total
Line 190, ppm, change to, mg/L
Line 190, methods of treating the adults need to be described in detail. How long time did you treat the adults before the collection of samples?
In Samples collection, feeding conditions and feed ingredients are introduced. I suggest adding sampling time, breeding generation and identification method, which may help to explain the high heterozygosity of the draft genome. Furthermore, I hope the authors describe why only the females were selected for sequencing and transcriptome analysis.
In the part of Methods, there is much software in this part which needs to be checked carefully. Some software lacks versions or literature information, such as "Juicer" in line 125 and "Juicebox" in 126 lack versions information, and in line 157 "Fasttree" lacks reference information.
In the part of Transcriptome analysis under phosphine fumigation/high temperature, there is no definite basis for phosphine concentration and temperature. This process may affect the number and type of differential expressed genes. I hope the author can explain why they choose this concentration and temperature.

Level of Interest

Please indicate how interesting you found the manuscript: Choose an item.

Quality of Written English

Please indicate the quality of language in the manuscript: Choose an item.

Declaration of Competing Interests

Please complete a declaration of competing interests, considering the following questions:

- Have you in the past five years received reimbursements, fees, funding, or salary from an organisation that may in any way gain or lose financially from the publication of this manuscript, either now or in the future?
- Do you hold any stocks or shares in an organisation that may in any way gain or lose financially from the publication of this manuscript, either now or in the future?
- Do you hold or are you currently applying for any patents relating to the content of the manuscript?
- Have you received reimbursements, fees, funding, or salary from an organization that holds or has applied for patents relating to the content of the manuscript?
- Do you have any other financial competing interests?
- Do you have any non-financial competing interests in relation to this paper?

If you can answer no to all of the above, write 'I declare that I have no competing interests' below. If your reply is yes to any, please give details below.

I declare that I have no competing interests

I agree to the open peer review policy of the journal. I understand that my name will be included on my report to the authors and, if the manuscript is accepted for publication, my named report including any attachments I upload will be posted on the website along with the authors' responses. I agree for my report to be made available under an Open Access Creative Commons CC-BY license (http://creativecommons.org/licenses/by/4.0/). I understand that any comments which I do not wish to be included in my named report can be included as confidential comments to the editors, which will not be published.

Choose an item.

To further support our reviewers, we have joined with Publons, where you can gain additional credit to further highlight your hard work (see: https://publons.com/journal/530/gigascience). On publication of this paper, your review will be automatically added to Publons, you can then choose whether or not to claim your Publons credit. I understand this statement.

Yes Choose an item.
